# Polarized localization of phosphatidylserine in the endothelium regulates Kir2.1

**DOI:** 10.1172/jci.insight.165715

**Published:** 2023-05-08

**Authors:** Claire A. Ruddiman, Richard Peckham, Melissa A. Luse, Yen-Lin Chen, Maniselvan Kuppusamy, Bruce A. Corliss, P. Jordan Hall, Chien-Jung Lin, Shayn M. Peirce, Swapnil K. Sonkusare, Robert P. Mecham, Jessica E. Wagenseil, Brant E. Isakson

**Affiliations:** 1 Robert M. Berne Cardiovascular Research Center,; 2 Department of Pharmacology,; 3 Department of Molecular Physiology and Biophysics, and; 4 Department of Biomedical Engineering, University of Virginia School of Engineering, Charlottesville, Virginia, USA.; 5 Division of Cardiology, Division of Medicine, SSM Health St. Louis University Hospital, St. Louis, Missouri, USA.; 6 Department of Cell Biology and Physiology, Washington University School of Medicine, St. Louis, Missouri, USA.; 7 Department of Mechanical Engineering and Materials Science, Washington University, St. Louis, Missouri, USA.

**Keywords:** Vascular Biology, Endothelial cells, Hypertension

## Abstract

Lipid regulation of ion channels is largely explored using in silico modeling with minimal experimentation in intact tissue; thus, the functional consequences of these predicted lipid-channel interactions within native cellular environments remain elusive. The goal of this study is to investigate how lipid regulation of endothelial Kir2.1 — an inwardly rectifying potassium channel that regulates membrane hyperpolarization — contributes to vasodilation in resistance arteries. First, we show that phosphatidylserine (PS) localizes to a specific subpopulation of myoendothelial junctions (MEJs), crucial signaling microdomains that regulate vasodilation in resistance arteries, and in silico data have implied that PS may compete with phosphatidylinositol 4,5-bisphosphate (PIP_2_) binding on Kir2.1. We found that Kir2.1-MEJs also contained PS, possibly indicating an interaction where PS regulates Kir2.1. Electrophysiology experiments on HEK cells demonstrate that PS blocks PIP_2_ activation of Kir2.1 and that addition of exogenous PS blocks PIP_2_-mediated Kir2.1 vasodilation in resistance arteries. Using a mouse model lacking canonical MEJs in resistance arteries (*Eln*^fl/fl^/Cdh5-Cre), PS localization in endothelium was disrupted and PIP_2_ activation of Kir2.1 was significantly increased. Taken together, our data suggest that PS enrichment to MEJs inhibits PIP_2_-mediated activation of Kir2.1 to tightly regulate changes in arterial diameter, and they demonstrate that the intracellular lipid localization within the endothelium is an important determinant of vascular function.

## Graphical abstract

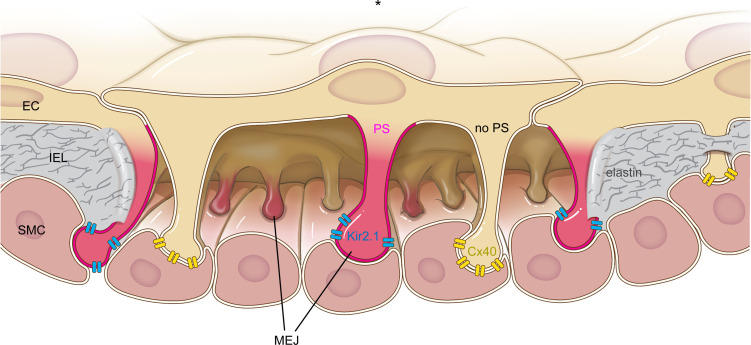

## Introduction

Kir2.1 is an inwardly rectifying potassium channel that maintains homeostatic potassium levels inside the cell (1). The channel is important for regulating cardiovascular homeostasis and is expressed in both the heart and arteries (2, 3). In resistance arteries, active currents have been observed in endothelium but not smooth muscle (3), and global heterozygous KO of Kir2.1 leads to hypertension in mice (2). In addition to its role in nitric oxide–based flow-mediated vasodilation (4), Kir2.1 is also a contributor to endothelial-derived hyperpolarization (EDH), the predominate dilation pathway in resistance arteries (3, 5, 6). Resistance artery endothelium contain a signaling microdomain called the myoendothelial junction (MEJ), which is an endothelial cell (EC) extension through holes in the internal elastic lamina (HIEL) that contacts the underlying smooth muscle cells (SMC). The MEJ is a critical site of heterocellular communication that facilitates EDH vasodilation through gap junctions and localization of proteins (7–9), such as the Ca^2+^-activated intermediate conductance potassium channel (IK_Ca_), Transient Receptor Potential Cation Channel Subfamily V Member 4 (TRPV4), and hemoglobin alpha (alpha globin) (5, 10–12). While functional data implicate that Kir2.1 may be at the MEJ, it has not been demonstrated. Furthermore, Kir2.1 channel function is known to be regulated by lipids, where cholesterol prevents normal activation (4, 13–16) and phosphatidylinositol 4,5-bisphosphate–Kir2.1 (PIP_2_-Kir2.1) interaction leads to channel opening and activation (17–21). Thus, we sought to determine if Kir2.1 was localized to the MEJ and how channel function was regulated by the local lipid environment.

We have previously demonstrated phosphatidylserine (PS) was enriched in MEJs isolated from a vascular cell coculture (VCCC) (22). PS is an anionic phospholipid enriched on the inner leaflet of lipid bilayers and comprises between 3% and 10% of the lipid composition of a cell (23). Given that PS has been implicated in regulating protein localization and inward-rectifying potassium channel function (24, 25), we sought to determine the contribution of PS to localization and function of Kir2.1 in resistance arteries. While some data indicate PS as a coactivator of Kir2.1 function (26, 27), in silico models demonstrate that PS can also bind to the PIP_2_ activation site (24, 28), implying that PS may be capable of preventing or competing with the necessary binding of PIP_2_ (18, 19) for Kir2.1 channel activity. We therefore hypothesized PS and Kir2.1 localized together at a unique subpopulation of MEJs, where PS could negatively regulate PIP_2_ activation of Kir2.1 and, thus, modulate the magnitude of vasodilation and total peripheral resistance.

## Results

### Spatial distribution and heterogeneity of MEJs.

**Figure 1:**
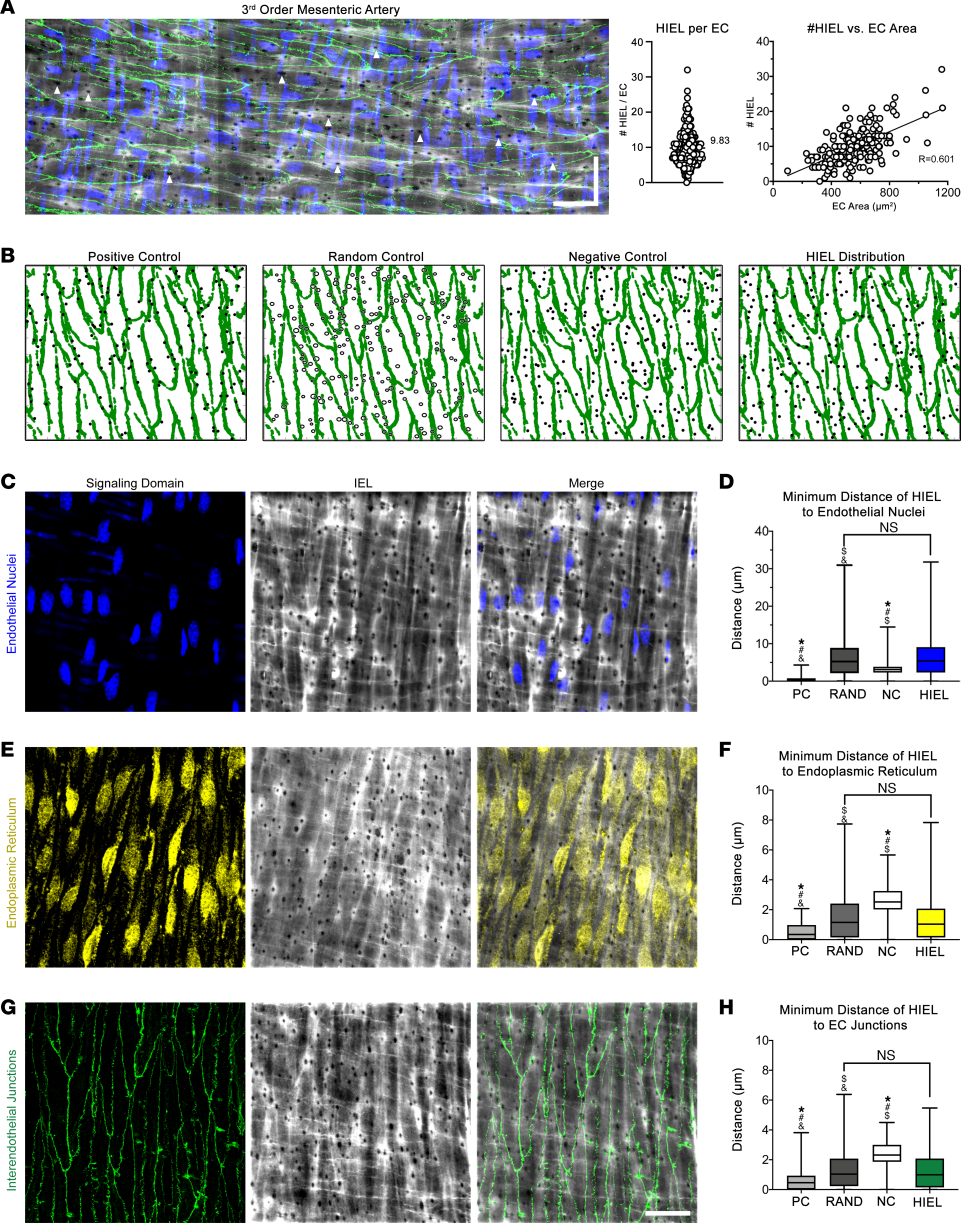
HIEL are randomly distributed with respect to endothelial signaling hubs. For all images, nuclei are detected via DAPI (blue), IEL detected via Alexa Fluor 488–linked hydrazide (gray), interendothelial junctions detected via claudin-5 (green), and endoplasmic reticulum detected via calnexin (yellow). (**A**) Representative stitched confocal image of a third-order mesenteric artery prepared en face. Scale bar: 30 μm in both directions. Quantification of HIEL per EC and plot of HIEL per EC versus EC area. *n* = 4 mice, *n* = 4 arteries, *n* = 12 ROIs, and *n* = 205 ECs. (**B**) Example graphical outputs of interendothelial junction thresholding via Matlab (green) and real-world or simulated HIEL (black). Random simulations incorporated variations in HIEL diameter while PC and NC simulations assumed uniform HIEL size (circles versus dots, respectively). (**C**) Representative en face confocal image of endothelial nuclei and IEL. (**D**) Box-and-whisker plot of minimum distance of real-world HIEL centers to endothelial nuclei compared with Matlab-simulated HIEL centers. *n* = 3 mice, *n* = 6 arteries, *n* = 18 ROIs, area = 9.75 × 10^4^ μm^2^, and *n* = 1,607 HIEL. (**E**) Representative en face confocal image of endoplasmic reticulum and IEL. (**F**) Box-and-whisker plot of minimum distance of real-world HIEL centers to endoplasmic reticulum compared with Matlab-simulated HIEL centers. *n* = 3 mice, *n* = 4 arteries, *n* = 9 ROIs, area=7.96 × 10^4^ μm^2^, and *n* = 1,157 HIEL. (**G**) Representative en face confocal image of interendothelial junctions and IEL. Scale bar: 30 µm. (**H**) Box-and-whisker plot of minimum distance of real-world HIEL centers to interendothelial junctions compared with Matlab-simulated HIEL centers. *n* = 6 mice, *n* = 10 arteries, *n* = 22 ROIs, area = 1.48 × 10^5^ μm^2^, and *n* = 2166 HIEL. Brown-Forsythe and Welch 1-way ANOVA, where ^#^*P* < 0.0001 significant difference to real-world HIEL distribution, *P < 0.0001 significant difference to random distribution, ^$^*P* < 0.0001 significant difference to negative control distribution, and ^&^*P* < 0.0001 significant difference to positive control distribution.

When arteries are cut longitudinally with the endothelial monolayer facing upwards (en face view), potential sites of MEJ formation are detected as HIEL (5, 29, 30). IHC studies on en face views have reported heterogeneous protein localization to the HIEL (29–37), with a possible juxtaposition to other signaling hubs in the endothelium. To determine if this may be the case, we initially used stitched confocal images of third-order mesenteric arteries stained for nuclei, the IEL, and interendothelial junctions (e.g., claudin-5) and visualized approximately 50 fully in-view EC per artery (Figure 1A). We found 9.83 HIEL per EC (Figure 1A), and the number of HIEL could be predicted by the size of an EC (Figure 1A), indicating that there may be a specific spatial pattern of HIEL. We developed a Matlab program (https://github.com/claireruddiman/Spatial-Distribution) to calculate the minimum distance of each individual HIEL to organelles and/or critical signaling hubs within EC and compared it against simulations for a positive, negative, and random spatial pattern (Figure 1B). We found a significant difference between real-world and simulated positive and negative values but no differences between real-world and simulated random values in all tested conditions; nuclei (Figure 1, C and D), endoplasmic reticulum (ER) (Figure 1, E and F), and interendothelial junctions (Figure 1, G and H). Descriptive statistics corresponding to box-and-whisker plots are included in Supplemental Table 1 (supplemental material available online with this article; https://doi.org/10.1172/jci.insight.165715DS1). The random distribution of HIEL was also evident in first-order mesenteric arteries where HIEL density is approximately half of the density in third-order arteries (Supplemental Figure 1), indicating that the random localization of the HIEL is not an outcome of high density. Thus, HIELs are randomly distributed with respect to major EC-signaling hubs.

**Figure 2:**
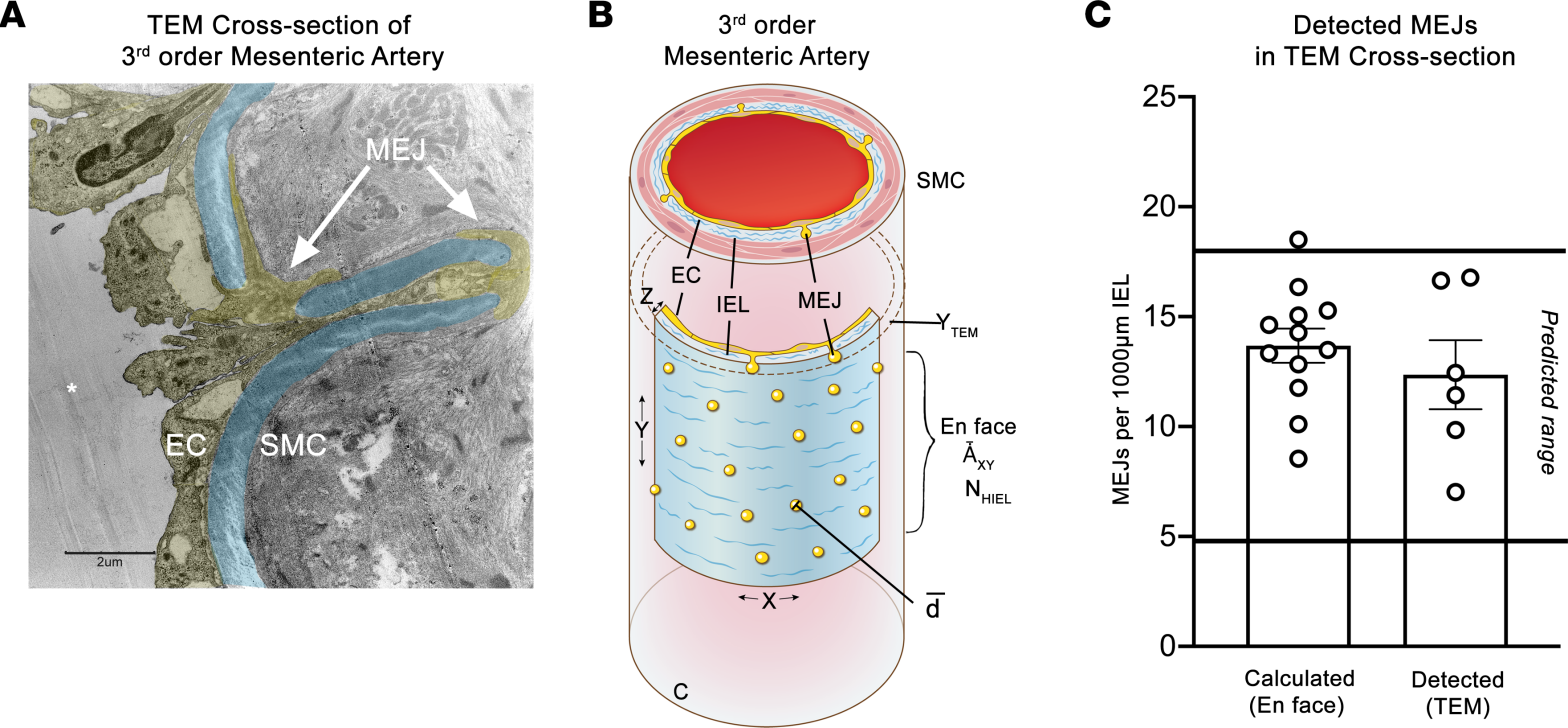
Every HIEL contains an MEJ. (**A**) Transmission electron microscopy (TEM) image of an arterial cross-section at 2,000× magnification. IEL is pseudocolored in blue, and ECs/MEJs are pseudocolored in yellow. Scale bar: 2 μm. (**B**) Quantitation of en face HIEL from Matlab simulations that were used to predict if every HIEL contains an MEJ (Supplemental Methods and Supplemental Tables 1–3). Where C is the circumference of the artery, d is the diameter of HIEL, Ǡ_xy_ is the area of en face images, N_HIEL_ is the average number of HIEL per image, Y_TEM_ is the thickness of an individual TEM section, X is the width of an en face image, Y is the height of an en face image, and Z is the thickness of the arterial wall. (**C**) Quantification of HIEL per 1,000 μm measured in TEM cross-sections or back calculated from en face views, where both values are within the predicted range. For TEM, *n* = 6 mice, *n* = 6 arteries, *n* = 3–5 TEM sections, and 570–964 μm IEL length quantified per mouse. For en face, *n* = 4 mice, *n* = 4 arteries, *n* = 12 ROIs, and area = 1.67 × 10^5^μm^2^.

Given the heterogeneity of proteins observed at the HIEL (30–37), if the HIEL were not specifically localized to EC signaling hubs as demonstrated in Figure 1, another alternative may be that not every HIEL contains an EC projection. We used TEM to examine transverse sections of third-order mesenteric arteries where cellular projections can be unequivocally identified at the ultrastructural level, thus circumventing the need to rely on an individual protein marker (Figure 2A). We leveraged our en face data obtained from our Matlab program and measurements taken from third-order mesenteric arteries (Figure 2B and Supplemental Tables 2 and 3) to predict how many MEJs would be observed in transverse TEM images (Supplemental Figure 3 and Supplemental Tables 2–4, Supplemental Methods). Based on our calculations, we predicted between 5 and 17.8 HIEL per 1,000 μm IEL length in the cross-sectional TEM view if all the HIEL were MEJs. Converting the HIEL density in en face images to HIEL per 1,000 μm IEL length falls within this predicted range, validating our prediction (Figure 2C). The measurements taken from TEM cross-sections also fall within this predicted range, where every HIEL contained a cellular projection (Figure 2C); thus, we conclude each HIEL contains a bona fide MEJ.

### PS and Kir2.1 define a unique subpopulation of MEJs.

**Figure 3:**
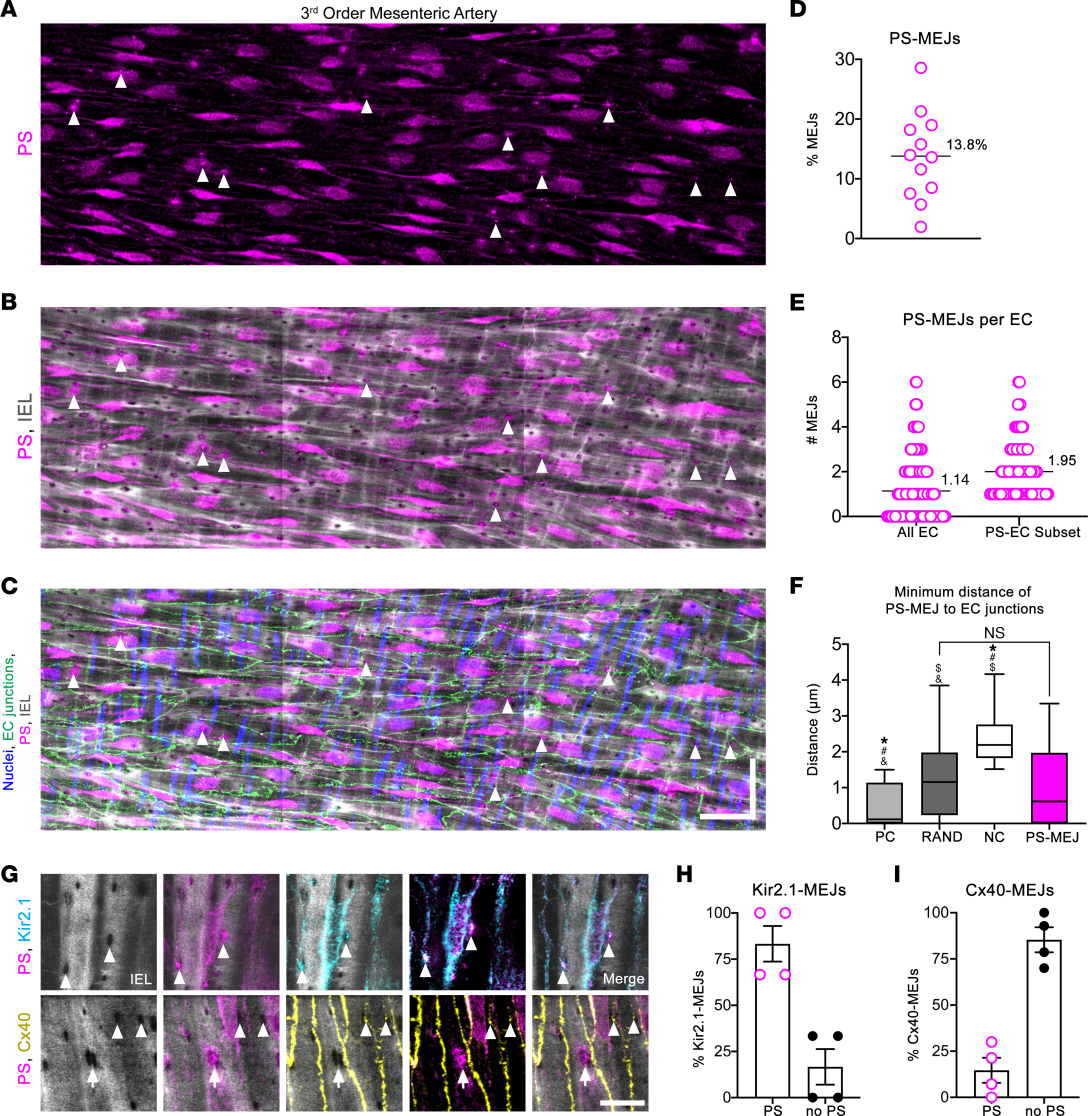
PS colocalizes with Kir2.1 in a subpopulation of MEJs. (**A**–**C**) Representative stitched confocal image of a third-order mesenteric artery prepared en face and stained for PS (magenta) (**A**), PS and IEL (gray) detected via Alexa Fluor 488-linked hydrazide (**B**), and merge with nuclei (blue) detected via DAPI and interendothelial junctions (green) detected via claudin-5 (**C**). Arrowheads show PS localized to MEJ. Scale bar: 30 μm in both directions. (**D**) Percentage of MEJs in endothelium containing PS. (**E**) Number of MEJs containing PS per EC, where EC borders were defined via claudin-5 staining. *n* = 4 mice, *n* = 4 arteries, *n* = 12 ROIs, and *n* = 205 ECs. (**F**) Spatial analysis of PS-MEJ compared with positive control (PC), random (RAND), and negative control (NC) simulations. Brown-Forsythe and Welch ANOVA with Holm-Sidak multiple comparisons, where ^#^*P* < 0.0001 significant difference from real-world MEJ distribution, *P < 0.0001 significant difference from random simulation distribution, ^$^*P* < 0.0001 significant difference from PC simulation distribution, and ^&^*P* < 0.0001 significant difference from NC simulation distribution. *n* = 3 mice, *n* = 4 arteries, *n* = 4 ROIs, *n* = 66 PS-MEJs, and area = 4.49 × 10^4^ μm^2^. (**G**) En face images of third-order mesenteric arteries with IEL (gray), PS (magenta), Kir2.1 (cyan), and Cx40 (yellow). Scale bar: 10 μm. Arrowheads indicate colocalization of PS and Kir2.1 to MEJ or only Cx40 to MEJ, and arrow indicates localization of only PS to MEJ. (**H** and **I**) In-house Matlab analysis to detect incidence of PS and Kir2.1 or PS and Cx40 colocalization to MEJ in en face images. *n* = 4 mice and *n* = 4 arteries per group.

If every HIEL contains an MEJ, it was unclear to us what contributes to the heterogenous localization of proteins to the MEJ, especially if there is a random distribution of MEJs with respect to EC signaling hubs. The possibility we considered was the lipid composition of MEJs, due to the emerging evidence of lipid regulation of proteins (17, 18, 24, 38). In vitro, we have previously demonstrated enriched PS via lipid mass spectrometry at MEJs isolated from VCCC (Supplemental Figure 3, Supplemental Methods; refs. 7, 22, 39), and we hypothesized the same lipid accumulation at in vivo MEJs. Stitched confocal images of third-order mesenteric arteries viewed en face revealed PS localization both to the ER where it is synthesized (40) and to MEJs in intact arteries (Figure 3, A–C). The specificity of the PS antibody was confirmed using a Lactadherin-C2 plasmid in HeLa cells, a protein that specifically binds to PS (Supplemental Figure 3 and Supplemental Methods) (41–44). PS was found to be heterogeneously distributed and occupied 13.8% of MEJs (Figure 3D), in contrast to the well-established MEJ-resident protein alpha globin, which occupies 40.39% of MEJs (Supplemental Figure 4). Analysis of MEJs containing PS (PS-MEJs) per EC reveals, on average, 1.14 PS-MEJs are associated with any individual EC (100% of ECs analyzed; Figure 3E) or 1.95 PS-MEJs if only considering ECs containing at least 1 (58.04% of all ECs analyzed Figure 3E). The PS-MEJs did not exhibit a localization pattern within EC (Figure 3F and Supplemental Table 1).

Because it is well known that lipid interactions with ion channels regulate channel activity (17, 18, 24, 38), we wanted to investigate if PS could have a similar function at the MEJ. In silico data suggest that PS has a binding site on Kir2.1 (24, 28), an important potassium channel involved in vasodilation (3), demonstrating that PS can access and bind the PIP_2_ binding site, possibly interfering with a necessary component of Kir2.1 activation (17–21). Using en face IHC, we observed that 83.33% of Kir2.1-MEJ puncta also contained PS (Figure 3, G and H; Supplemental Figure 5; and Supplemental Methods). In contrast, localization with PS occurred in only 14.64% of connexin 40–MEJs (Cx40-MEJs), a gap junctional protein known to facilitate EC and SMC communication in arteries (Figure 3I, Supplemental Figure 6, and Supplemental Methods; ref. 45). Thus, Kir2.1 is highly enriched in PS-MEJs, whereas Cx40 is segregated to non–PS-MEJs.

### PS localization to the MEJ dampens PIP_2_ activation of Kir2.1.

**Figure 4:**
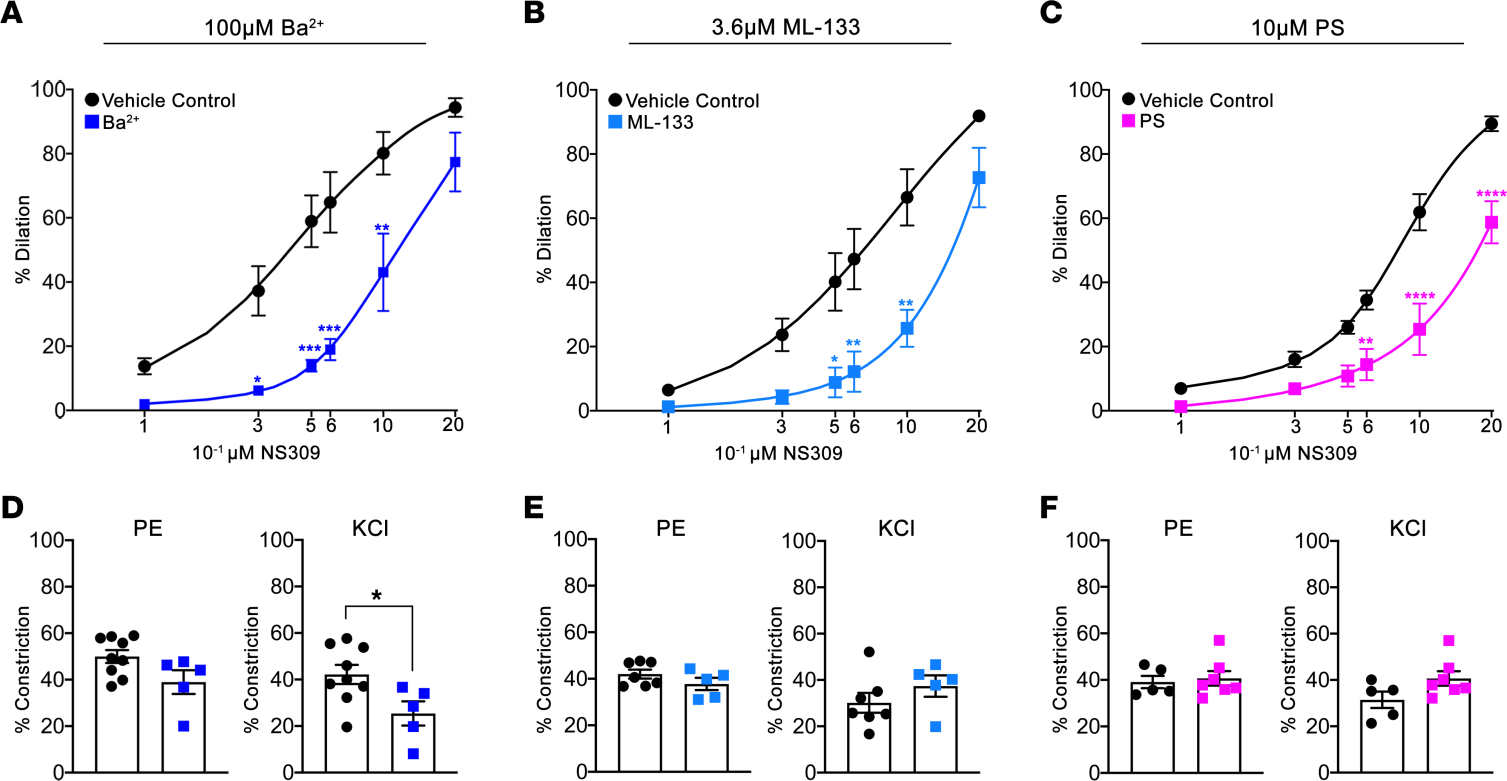
Exogenous PS blocks Kir2.1-mediated vasodilation. (**A** and **B**) Dose-response curves to NS309 on third-order mesenteric arteries in the presence of 100 μM Ba^2+^ (**A**), 3.6 μM ML-133 (**B**), or 10 μM PS (**C**) compared with vehicle controls of water, DMSO, and ethanol, respectively. A repeated measures 2-way ANOVA was performed, and significance differences were detected between treatment groups: of *P* < 0.0001, *P* < 0.005, and *P* < 0.0001, respectively. Sidak’s multiple comparison post hoc analysis was performed at each dose where *P < 0.05, **P < 0.01, ***P < 0.005, and ****P < 0.001. (**D**–**F**) Percent constriction to 1 μM PE or 30 mM KCl in the presence of each Kir2.1 inhibitor. Student’s *t* test was used, where *P < 0.05.

The strong colocalization of Kir2.1 and PS at MEJs, with in silico data indicating a PS binding site (24, 28), led us to hypothesize that PS may regulate Kir2.1 function. We wanted to test if PS could regulate Kir2.1 activation in third-order mesenteric arteries where Kir2.1 and PS localize to the same MEJs. We used pressure myography to perform dose response curves with NS309, a potent dilator that can activate Kir2.1 (3). We observed a reduced dilation to NS309 in the presence of known Kir2.1 inhibitors barium (Ba^2+^) (46, 47) at 100 μM (Figure 4A) and ML-133 (48, 49) at 3.6 μM (Figure 4B), verifying previously published results (3). We applied exogenous PS at 10 μM to the arteries and observed a similar reduction in NS309-mediated vasodilation (Figure 4C). We confirmed in this experimental setup that PS could get to the MEJ (Supplemental Figure 7 and Supplemental methods). Importantly, the application of PS or known Kir2.1 inhibitors did not influence SMC function (Figure 4, D–F); however, Ba^2+^ significantly inhibited KCl constriction, likely due to off-target effects on SMC Kir6.1 (50).

**Figure 5:**
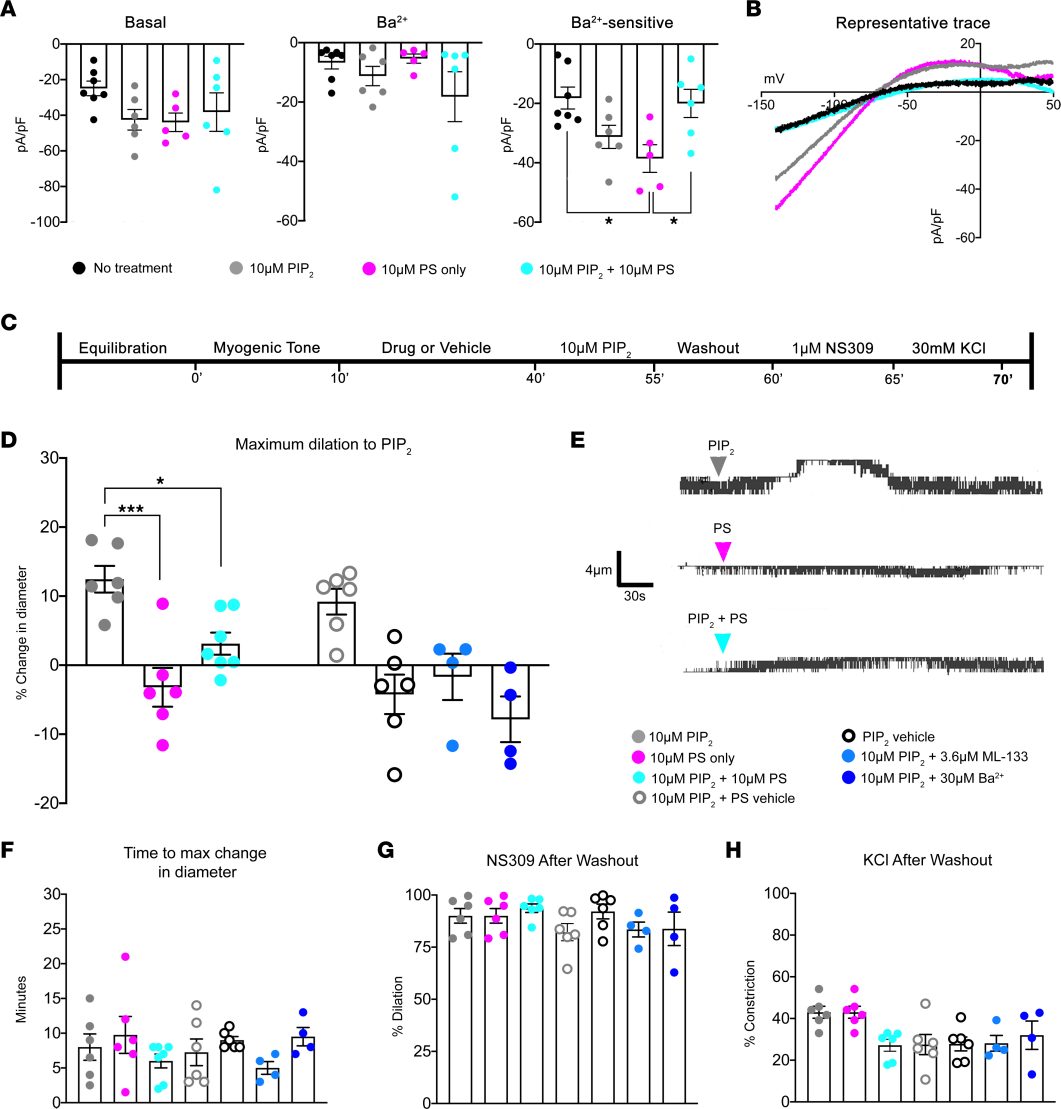
PS inhibits PIP_2_ activation of Kir2.1. (**A**) Average whole cell Kir2.1 currents at –140 mV in transfected HEK293T cells at baseline or with 100 μM Ba^2+^. Ba^2+^-sensitive currents were calculated by subtracting Ba^2+^ current from basal current. *n* = 5–7 cells per group. An ordinary 1-way ANOVA was performed with *P* < 0.05 significant effect for Ba^2+^-sensitive currents. Tukey’s multiple-comparison test was performed, where *P < 0.05. (**B**) Representative traces of Ba^2+^-sensitive currents, where basal current (black), 10 μM PIP_2_ (gray), 10 μM PS (magenta), and 10 μM PIP_2_ + 10μM PS (cyan). (**C**) Experimental timeline to assess PIP_2_ dilation in intact arteries. (**D**) Maximum change in diameter for each treatment group. *n* = 4–5 mice and *n* = 4–7 arteries per group. Changes in diameter for PS were taken from the same experiments as 10 μM PIP_2_ + 10 μM PS groups, where the maximum change in diameter was recorded within the 30-minute incubation prior to PIP_2_ treatment. An ordinary 1-way ANOVA was performed between PIP_2_, PS only, and PIP_2_ + PS with a significant interaction of *P* < 0.0005. Tukey’s multiple-comparison test was performed post hoc, where *P < 0.050 and ***P < 0.001. Other treatment groups were performed to ensure no unintended effects occurred with vehicles and to verify efficacy of Kir2.1 inhibitors; thus, they were excluded from the ANOVA. (**E**) Representative traces of inner diameter from pressure myography experiments treated with 10 μM PIP_2_, 10 μM PS, or 10 μM PIP_2_ + 10 μM. (**F**) Time point where maximum change in diameter was measured. (**G**) Dilation to 1 μM NS309 following a 5-minute washout period to assess EC function. (**H**) Constriction to 30 mM KCl to assess SMC function. One-way ANOVA was performed for **F**–**H**.

Next, using whole-cell patch clamp electrophysiology and HEK293T cells overexpressing Kir2.1, we tested the direct effect of PIP_2_, PS, or both on whole-cell currents (Figure 5, A and B). Average whole-cell currents are reported at –140 mV (Figure 5A), as commonly done in the field (51–53); for reference, we have included a comparison of average whole-cell currents at more positive, physiologically representative voltages in Supplemental Table 5. We observed increases in Ba^2+^-sensitive currents in both PIP_2_ and PS conditions alone (Figure 5A). Although the increase in Ba^2+^-sensitive currents with PIP_2_ is not significantly different from the control, the trend is consistent with previous observations of this lipid contributing to channel opening and activation (26, 27). Interestingly, when both PS and PIP_2_ are applied to the cells, the increase in Ba^2+^-sensitive currents was no longer observed (Figure 5, A and B), suggesting a dynamic interplay between the 2 lipids. Based on the inhibitory effect of PS on Kir2.1-mediated dilation in intact resistance arteries in Figure 4, and the well-characterized function of PIP_2_ on the Kir2.1 channel, our interpretation of the data is that PS can block the PIP_2_ activation of Kir2.1. Because there is strong evidence for PIP_2_-mediated dilation of Kir2.1 in cerebral arteries (17), we sought to test the effect of PS on PIP_2_-mediated dilation on arteries constricted to myogenic tone (Figure 5C). We observed a consistent dilation to 10 μM PIP_2_ in pressurized third-order mesenteric arteries that was blocked by Kir2.1 inhibitors Ba^2+^ and ML-133 (Figure 5, D and E). Surprisingly, PS treatment alone did not influence arterial diameter (Figure 5, B and C). In alignment with the electrophysiology data, we found that, when we preincubated the arteries with 10 μM exogenous PS for 30 minutes prior to adding PIP_2_, the dilation was significantly decreased (Figure 5, D and E) — an outcome cogent with PS competing for the PIP_2_ binding site (24, 28). The residual PIP_2_ dilation with a PS preincubation occurs at the same time as PIP_2_ alone, indicating that some PIP_2_ is able to access its binding site (Figure 5F), although to a significantly lower extent (Figure 5D). Following a washout period at the end of the experiment, arteries were evaluated for EC function via dilation to 1 μM NS309 (Figure 5G) and constriction to 30 mM KCl (Figure 5H) to ensure that measured differences in PIP_2_ dilation were not due to arteries of compromised function. These results indicate that the targeted localization of Kir2.1 to PS-MEJs contributes to channel regulation.

### Disrupted PS localization in endothelium increases PIP_2_ activation of Kir2.1.

**Figure 6:**
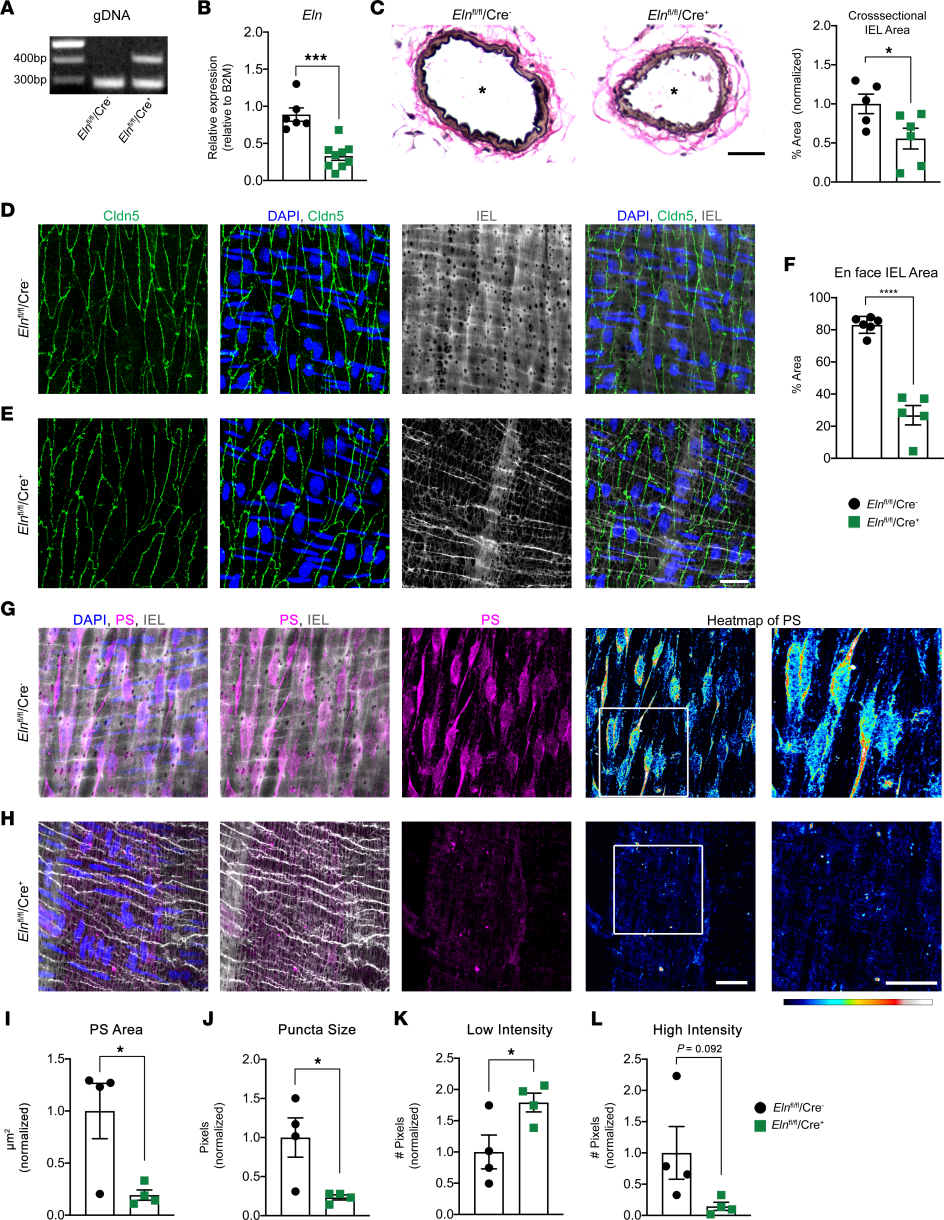
*Eln*^fl/fl^/Cre^+^ mice have disrupted PS localization in endothelium. (**A**) Genomic DNA (gDNA) isolated from EC-rich lung tissue demonstrating expected excision band of elastin in *Eln*^fl/fl^/Cre^+^ tissue at 410 bp. (**B**) qPCR on mesenteric vasculature to quantify mRNA levels of *Eln* for the 2 genotypes *Eln*^fl/fl^/Cre^–^ and *Eln*^fl/fl^/Cre^+^. *n* = 6–9 mice per group. Student’s *t* test; ***P = 0.0001. (**C**) Paraffin cross-sections of third-order mesenteric arteries stained with Verhoeff elastic stain (black) and van Gieson (pink), where the asterisk indicates lumen. Scale bar: 30 μm. Quantification of percent area occupied by Verhoeff stain on the right. *n* = 5–6 mice per group. Student’s *t* test; *P < 0.05. (**D** and **E**) En face IHC on *Eln*^fl/fl^/Cre^–^ and *Eln*^fl/fl^/Cre^+^ third-order mesenteric arteries where interendothelial junctions (green) are detected via claudin-5, nuclei (blue) are detected via DAPI, and IEL (gray) is detected via and Alexa Fluor 488–linked hydrazide. Scale bar: 30 μm. (**F**) Quantification of IEL area in en face views, expressed as a percentage of total image area. *n* = 5 mice, *n* = 5–6 arteries. Student’s *t* test; ****P < 0.0001. (**G** and **H**) En face IHC on *Eln*^fl/fl^/Cre^–^ and *Eln*^fl/fl^/Cre^+^ third-order mesenteric arteries where PS (magenta) and nuclei (blue) are detected via DAPI and IEL (gray) is detected via Alexa Fluor 488–linked hydrazide. PS heatmaps were generated using Royal Lookup Tables in ImageJ, where low-intensity signal is in cool tones (blue to cyan) and high-intensity signal is warm tones (yellow to white). The white box on the heatmap indicates the zoomed-in area shown to the right. Scale bars: 10 μm. (**I**–**L**) Quantification of PS area, puncta size, low-intensity pixels, and high-intensity pixels in PS en face images comparing *Eln*^fl/fl^/Cre^–^ and *Eln*^fl/fl^/Cre^+^ third-order mesenteric arteries. *n* = 3–4 mice, and *n* = 4–5 arteries per group. Student’s *t* test; *P < 0.05.

Next, we sought to determine if the MEJ itself was the key driver in PS-Kir2.1 organization and, by extension, vasodilation. To do this, we took advantage of floxed elastin mouse (*Eln*^fl/fl^*)* on an EC-specific Cre (Cdh5; i.e., *Eln*^fl/fl^/Cre^+^) that had previously been shown to lack IEL in resistance arteries (54) and, thus, possibly lacked MEJs. The mice had the correct elastin deletion and loss of IEL in resistance arteries (Figure 6, A–C; Supplemental Figure 8, A–C; and Supplemental Methods) but not large arteries (Supplemental Figure 8, D–G). The disruption of IEL was also evident in the en face view with a 56.26% reduction in hydrazide area and the loss of observable, morphologically distinct HIEL (Figure 6, D–F). Despite normal tight junction morphology (Figure 6E), the pattern of PS localization in endothelium is drastically changed (Figure 6, G and H), where it covers less surface area (Figure 6I), does not accumulate into large puncta (Figure 6J), and has decreased pixel intensity compared with controls (Figure 6, K and L).

**Figure 7:**
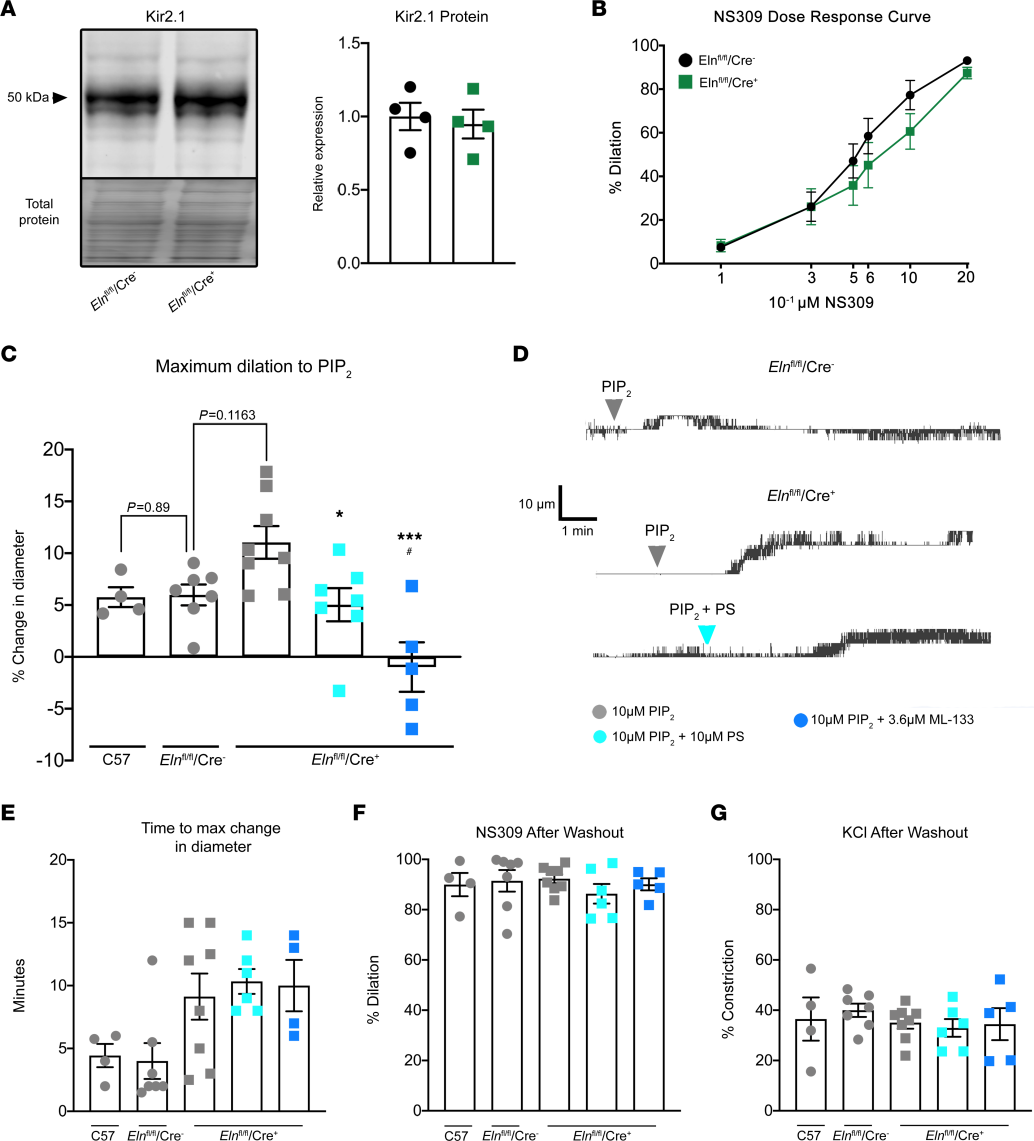
*Eln*^fl/fl^/Cre^+^ mice have increased dilation to PIP_2_. (**A**) Western blot on mesenteric vasculature to detect Kir2.1. Total protein was used as a loading control. Quantification was performed as a percentage of total protein and was then normalized with respect to the average protein expression in *Eln*^fl/fl^/Cre^–^ samples. *n* = 4 mice per group. Student’s *t* test was performed. (**B**) NS309 dose response curve on *Eln*^fl/fl^/Cre^–^ and *Eln*^fl/fl^/Cre^+^ third-order mesenteric arteries. *n* = 4 mice and *n* = 8–11 arteries per group. A repeated measures 2-way ANOVA was performed, and no significant differences were detected. (**C**) Dilation to 10 μM PIP_2_ at 60 mmHg. *n* = 4–5 mice, *n* = 4–8 arteries per group. A repeated-measures 2-way ANOVA was performed, and a significant effect of *P* = 0.0005 was detected. Tukey’s multiple comparison post hoc test was performed with *P < 0.05 in comparison with 10 μM PIP_2_ *Eln*^fl/fl^/Cre^+^, ***P < 0.005 in comparison with 10μM PIP_2_ *Eln*^fl/fl^/Cre^+^, and ^#^*P* < 0.05 in comparison with 10 μM PIP_2_ *Eln*^fl/fl^/Cre^–^. The 10 μM PIP_2_ C57 experimental group was used to control for a 60 mmHg experimental pressure and was therefore excluded from ANOVA. A Student’s *t* test was performed between 10 μM PIP_2_ C57 and *Eln*^fl/fl^/Cre^–^ groups, with no significance detected. (**D**) Representative traces from pressure myography experiments demonstrating dilation to PIP_2_ in *Eln*^fl/fl^/Cre^–^ arteries and *Eln*^fl/fl^/Cre^+^ arteries with or without a 30-minute PS preincubation. (**E**) The time point at which maximum change in diameter was achieved for each group. (**F**) Dilation to 1 μM NS309 following a 5-minute washout period to assess EC function in each experiment. (**G**) Constriction to 30 mM KCl to assess SMC function in each experiment.

In *Eln*^fl/fl^/Cre^+^ mice, Kir2.1 protein and function were unchanged as assessed by quantitative Western blot and NS309-mediated dilation, respectively (Figure 7, A and B). Given these results, if the PS polarization to MEJs were important for dampening Kir2.1-mediated dilation, application of PIP_2_ would have increased dilation in *Eln*^fl/fl^/Cre^+^ arteries. *Eln*^fl/fl^/Cre^–^ arteries were used at 60 mmHg based on their myogenic tone (Figure 7C and Supplemental Figure 9). This dilation was not different compared with arteries from C57BL/6 mice at 60 mmHg (Figure 7C). *Eln*^fl/fl^/Cre^+^ arteries exhibited a significant increase in PIP_2_-mediated vasodilation, which was inhibited with addition of 10 μM PS or 3.6 μM ML-133 (Figure 7C). Interestingly, arteries from *Eln*^fl/fl^/Cre^+^ had an increased time to maximum change in diameter (Figure 7, D and E) compared with *Eln*^fl/fl^/Cre^–^ or C67BL/6 arteries, which is reflective of a continued dilation in *Eln*^fl/fl^/Cre^+^ arteries compared with a transient dilation in control experiments. A continued dilation coincides with PS not being spatially oriented to negatively regulate the PIP_2_-mediated activation of Kir2.1 in *Eln*^fl/fl^/Cre^+^ arteries, thus resulting in an uncontrolled dilation. After a washout period, EC and SMC function were evaluated with 1 μM NS309 and 30 mM KCl, respectively (Figure 7, F and G).

## Discussion

### Subpopulations of MEJs have distinct functions.

**Figure 8:**
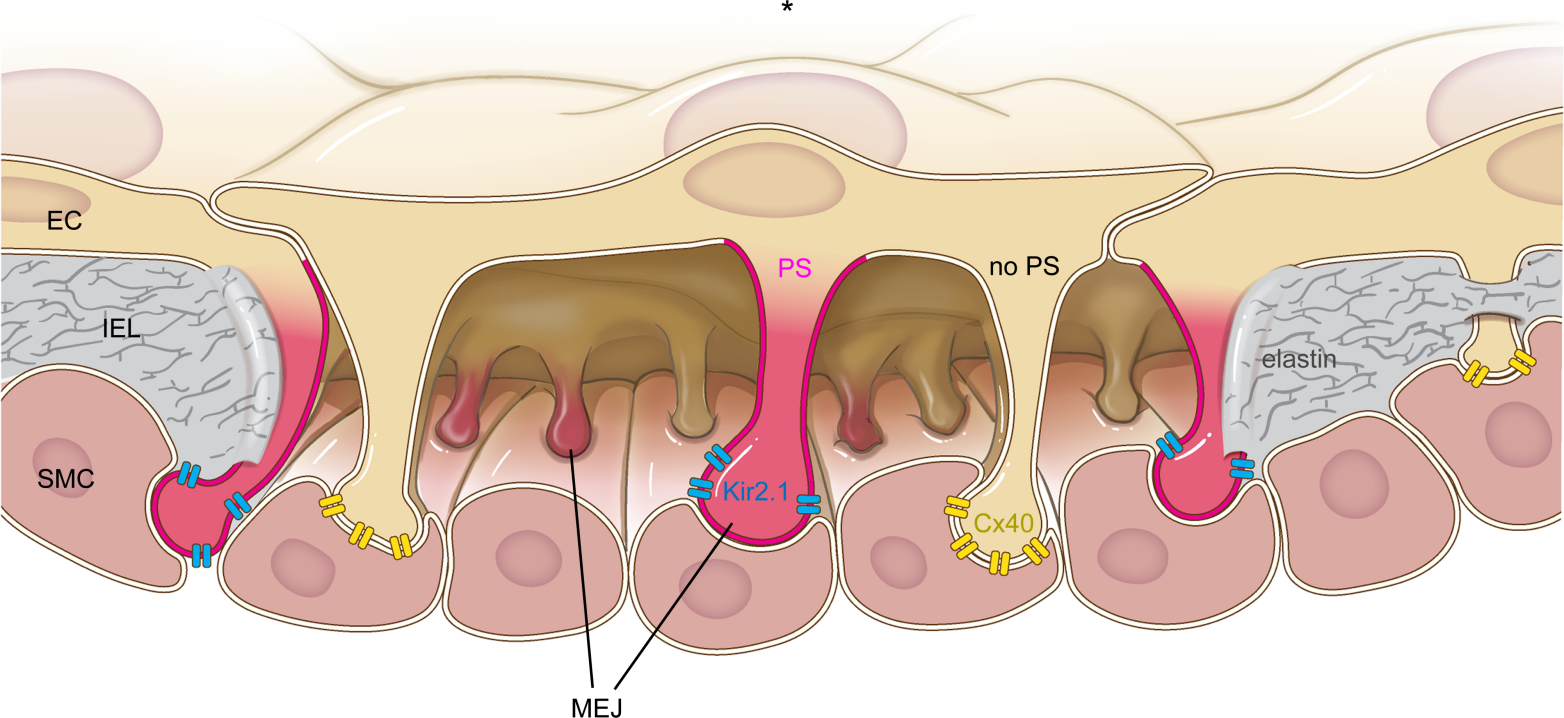
Heterogeneous lipid localization to MEJs results in functionally distinct subpopulations. A zoomed-in view of MEJs in the arterial wall, where the asterisk indicates the lumen, ECs depicted in beige along the lumen, SMCs shown in red along the bottom of the image, and MEJs between the 2 cell types and encased in IEL proteins such as elastin (gray). Kir2.1 channels (blue) localize to MEJs enriched with PS (magenta), while the gap junction protein Cx40 (yellow) localizes to MEJs without PS enrichment (beige). Image demonstrates how subpopulations of MEJs exist within the artery and have unique, specific functions. For clarity, the IEL is only shown at the edges of the image.

Here we have demonstrated that PS defines a functionally distinct subpopulation of MEJs where it localizes with and negatively regulates Kir2.1 through preventing PIP_2_ activation and is spatially separated from MEJs containing the gap junction protein Cx40 (Figure 8). The primary function of PS-Kir2.1 MEJs is likely to generate vasodilatory hyperpolarization, which can be communicated to SMCs via nearby gap junction MEJs. Segregating the generators and communicators of hyperpolarization to distinct subpopulations of MEJs (Figure 8) represents an important mechanism to tightly regulate resistance arterial diameter. This observation, coupled with the functional data herein, strongly suggests that heterogenous MEJ populations may exist to carry out unique functions: either to direct heterocellular communication or function in a vasodilatory capacity. Our data define a unique MEJ subpopulation, suggesting a role for PS in modulating PIP_2_ activation of Kir2.1 within the artery.

Another MEJ population that has been described is the A-kinase anchoring protein 150 (AKAP150)-TRPV4 MEJ, where AKAP150 is required for adequate activation of TRPV4 activation and vasodilation (10). This colocalization at the MEJ is important for facilitating the EDH pathway (11). In a separate study on rat resistance arteries, Cx37 and the IK_Ca_ colocalize in a subpopulation of MEJ, suggesting some potential for potassium channel and gap junction MEJ overlap (32). Further spatial and functional analysis of the Cx37-IK_Ca_ would be necessary to fully understand the role for the colocalization, but we speculate that the Cx37-IK_Ca_ MEJ population identified in rat resistance arteries may exist in small groups of ECs and correlate with heterogenous responses of ECs to receptor agonism (55). Interestingly, the Cx37-IK_Ca_ population contrasts with the spatial separation of Kir2.1 and Cx40 that we demonstrate here. However, it is not surprising that there is a difference in colocalization with gap junctional MEJs between these 2 potassium channels because of the differences in regulation, activation, and potassium flux. It is unknown what percentage of total MEJs that the AKAP150-TRPV4 or Cx37-IK_Ca_ populations occupy. The data presented in this paper include rigorous quantification of PS localization to MEJs and evaluation of spatial pattern within the endothelium. Future efforts to understand MEJ heterogeneity should use similar quantification methods for understanding possible coordinated function of unique subpopulations.

In our en face IHC PS localizes to the perinuclear region (Figure 3), suggesting localization in the ER. Given the distinct subpopulation of MEJs that PS occupies, there is likely a specific mechanism of PS transport to facilitate accumulation in the MEJ. Energy-driven lipid transport within a cell occurs via specific lipid transport proteins (LTP) at membrane contact sites, where 2 lipids are transported in opposite directions. A family of LTPs, called oxysterol-binding proteins (ORPs), have been implicated in the trafficking of PS to the plasma membrane (56–58). It could be that the expression of an ORP in ECs drives deposition of PS to specific the MEJs. Kir2.1 trafficking through the ER is also well described, and indeed, we see perinuclear staining in Kir2.1 en face preparations (Figure 3G) (59, 60). Given the specific, targeted localization of Kir2.1 to PS-enriched MEJs, we speculate that PS could also function in trafficking Kir2.1 to these signaling microdomains.

### PS regulation of Kir2.1.

Kir2.1 channel function is dependent on the presence of both PIP_2_ and an anionic phospholipid (26, 27). We have reproduced these data and have shown that both PS and PIP_2_ can activate the channel in an electrophysiology experiment. However, when added together, the lipids did not have an additive effect on channel activity; rather, they did not increase current beyond baseline (Figure 5, A and B). In agreement with this finding, our pressure myography data show that addition of exogenous PS blocks PIP_2_ activation of Kir2.1, suggesting that the role of PS at the MEJ may be primarily to inhibit Kir2.1 channel activity as opposed to activating the channel. Our data also demonstrate that exogenous application of PS inhibits agonist-mediated Kir2.1 activation in intact arteries via NS309, without affecting SMC constriction, reinforcing the idea of PS as a negative regulator of EC-mediated vasodilation in this tissue. Because potassium flux through Kir2.1 ultimately leads to vasodilation, the purpose of PS colocalization with Kir2.1 at the MEJ could be to dampen the channel’s K^+^ flux to prevent overshooting dilation in these resistance arteries. While we did not investigate this interaction in other arterial beds or sizes, we speculate that it could be maintained in small arteries throughout the body to tightly regulate vasodilation, especially in arteries where fluctuations in arterial diameter impact whole-animal physiology (cerebral or skeletal muscle vascular beds). Lastly, since EDH predominates in our model system (resistance arteries), we did not evaluate how PS influences Ach-mediated vasodilation; however, based on previous research (3), we speculate a similar inhibitory effect, and future investigation in larger arteries will continue to contextualize the lipid regulation of Kir2.1. An SMC-based mechanism for PS inhibition is also unlikely, since SMC function was preserved in the presence of PS (Figure 4, E and F) and because there are no active Kir2.1 currents in SMCs from resistance arteries (3).

To our knowledge, crystallography-based evidence for a PIP_2_-mediated conformational change only exists for Kir2.1 (18). This, combined with the recent in silico evidence suggesting that PS may compete for the PIP_2_-binding site, led us to investigate the Kir2.1-PIP_2_-PS signaling axis in resistance arteries. There is also evidence indicating that other vascular potassium channels, including SK and K_ATP_, are regulated by PIP_2_ (61, 62), and these channels may be affected in our vasodilation studies where exogenous lipids are added to intact arteries. However, our accumulation of evidence obtained from diverse experimental approaches gives us confidence in concluding that PS inhibits Kir2.1-mediated vasodilation through an interaction at the MEJ. The characterization of additional PS-channel interactions within intact tissue should be investigated in the future.

It has been demonstrated that relative concentrations of lipids can have differential effects on channel function (26), and in MEJs where PS is localized, it is likely that the PS concentration is magnitudes higher than the local PIP_2_ concentration, such that PIP_2_ interactions with the channel are limited. Although the local concentration of PIP_2_ remains elusive, we speculate that PIP_2_ has a short half-life at the MEJ, given the importance of the PIP_2_ cleavage product, inositol-1,4,5-trisphosphate, in facilitating heterocellular signaling to the SMC and the enrichment of the second cleavage product, diacylglycerol, via lipid mass spectrometry (22, 63). Thus, local PS enrichment at the MEJ may be antagonizing the PIP_2_ requirement for channel opening and may explain why, in intact arteries, PS blocks PIP_2_ activation of Kir2.1. Together with the pressure myography results, it is evident that the balance between PS and PIP_2_ within the membrane can have differential effects on the protein. We conclude that a relatively higher PS concentration at the MEJ functions to negatively regulate Kir2.1 opening.

Our pressure myography data indicate that exogenous PS alone does not influence arterial diameter, which is unexpected when compared with the electrophysiology experiments where PS activates the channel. A possible reason for the differential results of PS influence on Kir2.1 could be the differences in cellular lipid composition of HEK293T cells and intact arteries (26). For example, the plasma membrane of ECs in intact arteries is complex, with signaling microdomains defined by protein and lipid composition. One example of this is the high density of caveolae in endothelium (64). Caveolae are crucial for plasma membrane organization and are also enriched with cholesterol, with some studies also suggesting specific localization of PS and PIP_2_ to caveolae (65, 66). It is possible that there is interplay between PS and cholesterol, a known depressor of Kir2.1 activity (4, 13, 14), in intact arteries that prevents PS alone from having a dilatory effect. Indeed, this could be another mechanism by which cholesterol prevents Kir2.1 activation. While we don’t specifically test whether the PS-Kir2.1 MEJ population also contains caveolae, it is highly likely that caveolae are present within this population, given the high density of caveolae in the endothelium and at MEJs (64, 67, 68). In the future, the interplay between cholesterol, PS, and PIP_2_ at the MEJ should be investigated in the context of Kir2.1 and other ion channels.

### The role of MEJ in facilitating PS regulation of Kir2.1.

We demonstrate the inhibitory effect of PS on PIP_2_ activation using arteries from a mouse model lacking typical HIEL morphology and, thus, MEJ formation (*Eln*^fl/fl^/Cre^+^). In this mouse, the PS localization within the intact resistance arterial endothelium is completely disrupted (Figure 6), covering less surface area and demonstrating reduced accumulation into punctate (reduced high-intensity signal). Notably, Kir2.1 protein expression is unchanged; however, *Eln*^fl/fl^/Cre^+^ arteries exhibited an increased dilation to PIP_2_, which was brought back down to control levels by adding PS to the artery. These results strongly support our findings of PS negatively regulating PIP_2_-mediated Kir2.1 vasodilation. Based on the result that the PIP_2_ dilation can be recovered to control levels with the addition of PS or ML-133 (a known Kir2.1 inhibitor) (49), we attribute the increase dilation to the increased access of PIP_2_ to Kir2.1. This increased dilation is likely due to the disrupted PS localization in the endothelium rather than a direct result of reduced elastin protein; this is cogent with Kir2.1 not demonstrating any known adhesion properties or being regulated by ECM proteins. However, there are some studies linking integrin signaling to ion channel function (69) and, in particular, increasing current through Kir2.1 (70). In *Eln*^fl/fl^/Cre^+^ resistance arteries, integrin signaling is likely decreased, since there is a reduction of integrin substrate (ECM proteins). Thus, integrins are an unlikely culprit because of an observed increase in PIP_2_-mediated dilation. Lastly, while myogenic tone was reduced in *Eln*^fl/fl^/Cre^+^ resistance arteries at high pressures (Supplemental Figure 9), vasodilation and constriction in *Eln*^fl/fl^/Cre^+^ resistance arteries were normal (Figure 7, E and F). To control for the mechanical differences in *Eln*^fl/fl^/Cre^–^ and *Eln*^fl/fl^/Cre^+^ arteries, we performed vasodilation experiments at 60 mmHg, where the myogenic tone is unchanged between the 2 groups (Supplemental Figure 9). These data lead us to conclude that lipid-induced phenotypes are independent of the myogenic tone phenotype.

### Limitations of our study.

Our interpretation of PS negatively regulating PIP_2_-mediated activation assumes local PIP_2_ concentration at the MEJ is orders of magnitude lower compared with PS. It is a technical challenge to establish the local lipid concentration of PIP_2_ at the MEJ; it was not detected in lipid mass spectrometry of in vitro MEJs; rather, a product of its cleavage, DAG, was found to be enriched at MEJ compared with EC or SMC (22). This suggests that the half-life of PIP_2_ at the MEJ is short lived because its cleavage products are needed for downstream vasodilatory signaling (22, 63). Even though we are unable to quantify the exact concentration of PIP_2_ at MEJs, our lipid mass spectrometry (22) and en face evidence (Figure 3) demonstrate a polarized enrichment of PS to a subpopulation of MEJs, suggesting an orders of magnitude higher concentration of PS at these MEJs compared with other lipids typically in the plasma membrane, including PIP_2_, thus reducing relative concentrations of these other lipids at this signaling domain. This paired with a conceivably short PIP_2_ half-life at the MEJ strongly suggests a minute local concentration of PIP_2_ and implies that Kir2.1 is surrounded by predominately PS at these signaling microdomains.

In this study, we use DOPS, a synthetically modified species more resistant to oxidative stress compared to PS derived directly from brain tissue. There are various PS species that differ mainly through geometrical and compositional differences in the tail group. Since the headgroup is the most critical component for the lipid-channel interaction (24), we do not predict minimally altered tail structures to interfere with expected channel interactions. However, we speculate that bulkier PS tail groups could hinder accessibility to the lipid binding site, either through changing the residence time of PS on Kir2.1 or limiting the number of lipid molecules capable of interacting simultaneously. Lipid structure and position within the membrane (71) can be altered by oxidative stress, a hallmark of cardiovascular diseases; therefore, the inhibitory effect of PS on Kir2.1 could either be enhanced or limited in disease states.

We present evidence of lipid regulation of Kir2.1 using an *Eln*^fl/fl^/Cre^+^ mouse model. It should be noted that this is the first study to our knowledge to investigate endothelial function on these mice. While ECs appear to function normally (Figure 7B), myogenic tone in resistance arteries (Supplemental Figure 9) is significantly altered at higher pressures with deviation from controls beginning at 80 mmHg, a pressure within normal physiological range. However, it is clear that the polarized localization of PS was lost in these arteries, and despite Kir2.1 protein expression and basal function remaining unchanged (Figure 7, A and B), the sensitivity to PIP_2_-mediated vasodilation was increased (Figure 7C), strongly supporting our evidence of MEJ-localized PS regulating Kir2.1 function. Thus, the increased sensitivity to PIP_2_ dilation is unlikely the result of altered myogenic tone.

### Outlook.

Our data demonstrate how spatial polarization of a lipid in native ECs can regulate the function of an ion channel, where channel activation by 1 lipid is modulated by the concentration of a second lipid. While lipid regulation of ion channels and proteins is well accepted, homogenous lipid distributions across the plasma membrane are often assumed, such as in simulations or liposome experiments, and are not representative of native lipid environments. Here we have demonstrated how intracellular spatial polarization of a lipid within intact tissue can selectively regulate the function of an ion channel for essential physiological function. Understanding the plasma membrane composition surrounding ion channels or proteins in their physiological environment could clarify results from lipid-protein interaction simulations, identify new lipid regulators, or explain tissue-specific function. Thus, investigation of lipid microenvironments in cells may offer a mechanism for differential ion channel function across tissues. Although more work needs to be done to understand how lipid dynamics affect ion channel function, the data presented herein provide a foundation for understanding ion channel regulation by compartmentalization of plasma membrane lipids.

## Methods

### Mice.

The elastin gene, *Eln*, was selectively knocked out of ECs via a VE-cadherin Cre (*Eln*^fl/fl^/Cdh5Cre^+^; ref. 54). These mice were on a C57BL/6 background, and both sexes within the age range of 10–20 weeks were used for experiments. For experiments on this KO mouse, *Eln*^fl/fl^/Cdh5Cre^–^ littermates were used. For all other experiments, C57BL/6J male mice between 10 and 20 weeks were used and purchased from Taconic. Mice were fed normal chow and housed under a 12-hour light/dark cycle. Mice were sacrificed via CO_2_ inhalation with a secondary method of cervical dislocation. All experiments were approved by the University of Virginia Animal Care and Use Committee.

### Antibodies and plasmids.

The following connexin plasmids were a gift from Janis Burt: pcDNA3.1 hygro mouse Cx37, pcDNA3.1 puro mouse Cx40, and pcDNA3 neo mouse Cx43. We purchased pEGFP-C1 neo Lactadherin-C2 (Addgene, 22852) and pCAG-Kir2.1-T2A-tdTomato (Addgene, 60598). pcDNA3.1 Panx1 was cloned in house.

Antibodies used were rabbit anti-PS (Biomatik, CA30389), mouse anti-KCNJ2 (MilliporeSigma, SAB5200027), mouse anti-Cx40 (Thermo Fisher Scientific, 37-890), goat anti-calnexin (Abcam, ab219644), and Alexa Fluor 488– or Alexa Fluor 647–linked hydrazide (Thermo Fisher Scientific, A10436 and A20502, respectively).

### En face IHC.

Mesenteric arteries were fixed with 4% paraformaldehyde at 4°C for 15 minutes. Following PBS washes, arteries were cut en face using microscissors and pinned out with tungsten wire (Electron Tube Store, 1439, 0.013 mm diameter) on small Sylgard squares (Electron Microscopy Sciences, 24236-10; dimensions, ~0.5 mm thick × ~1 cm tall × ~0.5 cm wide). Next, en face preps were permeabilized in 0.2% NP40/PBS at room temperature for 30 minutes, blocked with BSA or animal serum for 1 hour, and incubated with primary antibody 1:100 at 4°C overnight in blocking solution. Blocking solutions were either 5% serum or 10% BSA (for PS experiments) in 0.2% NP40/PBS.

The next day, en face preps were washed in PBS and incubated with secondary antibodies (1:400) at room temperature for 1 hour in blocking solution. The Alexa Fluor 488– or Alexa Fluor 647–linked hydrazide was included in the secondary antibody solution at a final dilution of 1:1,250–1:2,500 to visualize the IEL. Prior to dilutions for experiments, Alexa Fluor hydrazides stock solutions were made using diH_2_O at a concentration of 1 mM and stored at 4°C for up to 6 months. Next, the arteries were washed 3 times for 10 minutes in PBS. The third PBS wash included DAPI (Invitrogen, D1306) at 1:5,000 from a 5 mg/mL stock solution. Following PBS washes, the Sylgard square was lifted out of the plate, and the back was dried with a Kimwipe and then placed onto a microscope slide. A droplet of Prolong Gold with DAPI was dispensed on top of the Sylgard square (~20 μL, Invitrogen, P36931). Lab tape was used to secure a square coverslip to the microscope slide prior to imaging. Images were obtained on either a Zeiss 880 LSM with Airyscan (Matlab) or Olympus Fluoview 1000 with a 40× oil objective and 1.8 zoom.

### Analysis of stitched confocal en face images.

Stitched confocal images of arteries prepared en face were taken as 3 images on a Zeiss 880 LSM confocal microscope with a 40× objective and 1.8 zoom. Each individual image was analyzed using our in-house Matlab analysis program in order to identify HIEL and PS-HIEL. Next, individual ECs were traced using claudin-5 staining in ImageJ (NIH) and assigned a number. HIEL data obtained from Matlab were then manually organized by EC number in Microsoft Excel for analysis.

### Custom analysis of immunofluorescence en face images.

Briefly, each immunofluorescence channel of an en face image was subject to customized thresholding in order to detect HIEL and EC signaling hub (nuclei, interendothelial junctions, or ER, depending on experiment). The brightness and contrast settings were adjusted around difficult-to-detect HIEL in ImageJ prior to thresholding in Matlab. For claudin-5 experiments (not calnexin or nuclei), the ImageJ line tool was used to assist in detection of low-intensity claudin-5 signal at interendothelial junctions, and nonspecific background noise in the middle of the cell was removed to facilitate correct thresholding in Matlab program. Thresholding was confirmed as accurate through a visual output with thresholded area overlayed on original *Z* stack image.

This thresholding was used to calculate the minimum distance of HIEL center points to the EC signaling hub considered, and this distribution was plotted as a box-and-whiskers plot. The distribution was then compared with positive (PC), negative (NC), and random control (RAND, via Monte Carlo) simulations in order to determine the spatial distribution. PC simulations generate HIEL within 1 μm of signaling hub, NC generate HIEL between 1.5 and 4.5 μm of signaling hub, and RAND simulations generate randomly distributed HIEL. For PC and NC simulations, each HIEL simulated was the average diameter of all HIEL within the image. Representative plots in Figure 1B show HIEL center points of equal diameter. For RAND simulations, in addition to a random position being selected for the HIEL, a random HIEL diameter was chosen within the range of HIEL sizes measured for each image. A representative plot in Figure 1B shows RAND-simulated HIEL of varying diameter.

Puncta detection of PS, Kir2.1, and Cx40 was also done in this automated Matlab program, where a punctate was defined as being within an HIEL if its center point was within 0.75 μm of the HIEL center point. This definition was verified as accurate via a visual output from the program. The same PC, NC, and RAND simulations were run on PS-MEJs to determine spatial patterns. Data were transferred to GraphPrism, plotted as a box-and-whisker plot, and Brown-Forsythe and Welch ANOVA and Holm Sidak’s multiple comparisons statistical tests were performed to detect differences. Outliers were removed (ROUT outlier removal method with Q = 1%). *n* values for HIEL in figure legends include the entire set and do not reflect data points removed via outlier analysis. In-house Matlab code, example images, and instructions on how to use the code are available on GitHub (https://github.com/claireruddiman/Spatial-Distribution; commit ID: 34c3c1a).

### Transmission electron microscopy.

Third-order mesenteric arteries were dissected from mice and fixed in 4% paraformaldehyde/2.5% glutaraldehyde/PBS for a minimum of 4 hours. Arteries were then processed at the UVA Advanced Microscopy Facility. The arteries were washed in cacodylate buffer, incubated in 2% osmium tetroxide for 1 hour, washed in cacodylate buffer, and dehydrated with ethanol washes prior to incubation in 1:1 propylene oxide/epoxy resin (PO/EPON) overnight. The next day, the samples were incubated in a 1:2 PO/EPON mixture for 2 hours, a 1:4 PO/EPON mixture for 4 hours, and 100% EPON overnight. The next day, the samples were baked in an oven at 65°C prior to sectioning. Ultrathin 70 nm sections were mounted on a mesh copper grid. Sections were contrast stained with 0.25% lead citrate for 5 minutes, 2% uranyl acetate for 20 minutes, and then again 0.25% lead citrate for 5 minutes. Sections were visualized and imaged using a JEOL 1230 Transmission Electron Microscope. To avoid the possibility of double counting an HIEL, only 1 section per 5 μm artery length was analyzed, and a minimum of 500 μm of IEL length was analyzed per artery.

### Western blot.

Protein samples were loaded at 20 μg per well and run on an 8% gel (Invitrogen, WG1001BOX) at 170 V for 70 minutes. Protein was transferred to a nitrocellulose membrane (Genesee, 84-876) at 100 V for 1 hour. After a 20-minute wash with diH_2_O, total protein was visualized with Revert 700 Total Protein Stain (Licor, 926-11021) and imaged on a Licor Odyssey. Total protein stain was reversed by 2× rinses with reversal solution (0.1M NaOH/30% MeOH in diH_2_O). Next, the membrane was blocked for 1 hour in 5% BSA/TBS at room temperature with gentle rocking. Primary antibodies were applied at a 1:1,000 dilution in blocking solution overnight at 4°C with gentle rocking. The following day, after 2× 10-minute 0.02% Tween 20/TBS washes with medium shaking, a secondary antibody (Licor, 926-32210) was applied at a 1:10,000 dilution in blocking solution for 1 hour at room temperature with gentle shaking. Membranes were imaged on a Licor Odyssey. Quantification was done relative to total protein and normalized to the average value of controls.

### Whole-cell patch-clamp electrophysiology.

HEK293T cells at 60% confluence (10 cm dish) were transfected for 24 hours with 14 μg of pCAG-Kir2.1-T2A-tdTomato (Addgene, 60598) using 21.7 μL Lipofectamine 3000 (Thermo Fisher Scientific, L3000008) and 28 μL P3000 (Thermo Fisher Scientific, L3000008). Transfection was confirmed prior to experiment by tdTomato fluorescence using a confocal microscope epi-lamp. HEK293T cells were a gift from Douglas Bayliss (University of Virginia, Department of Pharmacology).

Currents were recorded in a conventional whole-cell patch clamp configuration with a voltage ramp from –140 mV to 50 mV over 250 ms and were measured with 10 μM diC8PIP_2_ (10 mM stock in Krebs-HEPES, Cayman Chemical, 64910), 10 μM PS (10.15 mg/mL stock in 1:1 EtOH/diH_2_O, Avanti, 840035), or 10 μM diC8PIP_2_ + 10 μM PS in pipette solution. For each of these conditions, Ba^2+^-sensitive currents were measured by adding 100 μM Ba^2+^ to the bath solution and by recording the current 5 minutes later. For basal measurements, the current was recorded after equilibration. The pipette solution consisted of 10 mM HEPES, 30 mM KCl, 10 mM NaCl, 110 mM K-aspartate, and 1 mM MgCl2 (adjusted to pH 7.2 with NaOH). HEPES-PSS was used as the bath solution (10 mM HEPES, 134 mM NaCl, 6 mM KCl, 1 mM MgCl_2_ hexahydrate, 2 mM CaCl_2_ dihydrate, and 7 mM dextrose, pH adjusted to 7.4 using 1M NaOH).

Patch electrodes were pulled with a Narishige PC-100 puller (Narishige International USA) and polished using a MicroForge MF-830 polisher (Narishige International USA). The pipette resistance was 3–5 ΩM. Data were acquired using a Multiclamp 700B amplifier connected to a Digidata 1550B system and analyzed using Clampfit 11.1 software (Molecular Devices).

### General pressure myography.

For all experiments, Krebs-HEPES buffer containing (in mM) NaCl 118.4, KCl 4.7, MgSO_4_ 1.2, NaHCO_3_ 4, KH_2_PO_4_ 1.2, HEPES 10, and glucose 6. On the day of the experiment, CaCl_2_ was added to the buffer at a final concentration of 2 mM. Buffer was pH measured on the day of the experiment and adjusted to be within 7.40–7.42 by adding NaOH or HCl. Mice were sacrificed using CO_2_ asphyxiation. The mesentery was dissected out and placed in ice-cold buffer and was then pinned out on a 10 cm plate filled with approximately 0.5 cm of Sylgard (Electron Microscopy Sciences, 24236-10; Fine Science Tools, 26002-10). Third-order mesenteric arteries, defined as the third branch point relative to the feed artery and with a maximum diameter between 100 and 200 μm, were cleared of surrounding adipose tissue using super fine forceps (Fine Science Tools, 11252-00) and microscissors (Fine Science Tools, 15000-03). The artery was then cut out of the mesentery, placed into the arteriograph chamber (DMT) containing Krebs-HEPES, and cannulated on glass pipette tips using super fine forceps (Fine Science Tools, 11254-20) and suture (DMT, Nylon, P/N 100115). Glass pipette tips were created from glass rods (World Precision Instruments, 1B120F-4) using a Narshige pipette puller (Model PC-100) with instrument settings A = 65 and B = 55. Buffer was gently pushed through the artery to clear remaining blood before cannulating the second side. Following cannulation, the artery was equilibrated over a 25-minute period by increasing the pressure from 20 to 80 mmHg in 20 mmHg increments (Big Ben Sphygmomanometer) and by slowly heating the artery to 37°C. For experiments on *Eln*^fl/fl^/Cre^–^ or *Eln*^fl/fl^/Cre^+^ mice, arteries were equilibrated to 60 mmHg. The buffer was circulated between the arteriography chamber and a beaker containing an excess reservoir of buffer by using a peristaltic pump (Atalyst Masterflex, FH30) to prevent overheating and to facilitate the delivery of pharmacological agents. DMT cell culture pressure arteriography setups were used, and the inner diameter of the vessel was recorded with the 2015 release of the DMT software.

For myogenic tone experiments, third-order arteries were cannulated, pressurized to 60 mmHg, and equilibrated to 37°C. After the development of myogenic tone, EC function was evaluated using 1–2 μM NS309. Maximum diameter was recorded 5 minutes later, and NS309 was washed out of the system until myogenic tone returned. The active curve was completed by increasing the pressure in 20 mmHg increments from 20 to 120 mmHg. Diameter was recorded after 5 minutes of plateaued diameter at each pressure (D_ACT_). After the active curve, pressure was brought back down to 60 mmHg for 5 minutes. SMC and EC function were evaluated with 10 μM PE dose, 10 μM Ach, and 30 mM KCl. Next, Ca^2+^-free Krebs-HEPES was circulated in the system for 15 minutes. The passive curve was then completed by increasing the pressure in 20 mmHg increments from 20 to 120 mmHg. Diameter was recorded after 5 minutes of equilibration at each pressure (D_PASS_). Myogenic tone was calculated using Equation 1.

 (Equation 1)
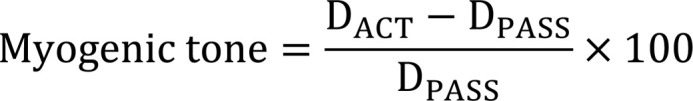


The percent change in diameter relative to 20 mmHg was calculated for active and passive curves using Equation 2, where D_X_ is diameter at any pressure and D_20_ is the diameter at 20 mmHg.

 (Equation 2)
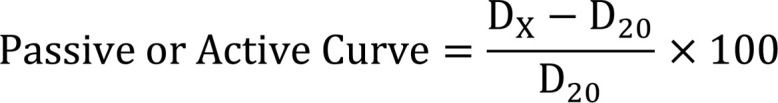


### NS309 dose response curves.

For NS309 curves, the total system volume was 50 mL to facilitate delivery of small doses of NS309. The artery was preconstricted using 1 μM phenylephrine (PE) in water (MilliporeSigma, P6126), and the inner diameter was recorded after 10 minutes (D_PE_). A 10 mM NS309 (MilliporeSigma, N8161) in DMSO stock solution was prepared, and 10 μL aliquots were stored at –20°C in amber microcentrifuge tubes. The stock solution was thawed prior to the experiment, and the following NS309 concentrations (in μM) were tested in the 50 mL system volume: 0.1, 0.3, 0.5, 0.6, 1, and 2, where the 0.1 μM dose corresponds to 0.5 μL of the NS309 stock (D_NS309_). The inner diameter was recorded as the average over a 7-minute time period for each dose of NS309. SMC function was assessed by adding 30 mM KCl (1M stock in water), and the inner diameter was recorded as the plateau after 5–10 minutes. The bath was then replaced with Ca^2+^-free Krebs-HEPES, containing 1 mM EGTA and 100 μM SNP, and the inner diameter was recorded after 10 minutes (D_MAX_). The data were exported to Microsoft Excel to calculate percent dilation using Equation 3.

(Equation 3)
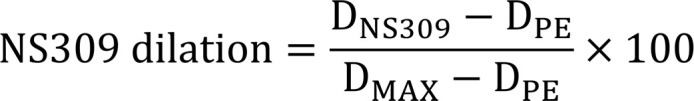


The percent vasodilation was plotted in GraphPad Prism as a dose response curve. A repeated measures 2-way ANOVA was performed between treatment groups, and multiple comparison post hoc analyses were subsequently performed. Details for statistical tests are included in each figure legend. For experiments where Kir2.1 was inhibited and where ML-133 hydrocholoride (MilliporeSigma, 422689, IC_50_ = 1.8μM) (49) was prepared as 7.2 mM stocks in DMSO and was added at a final concentration of 3.6 μM. For Ba^2+^ experiments (MilliporeSigma, 217565), the stock solution was prepared as 100 mM water and added at a final concentration of 30–100 μM. For PS experiments, stock solution was prepared as a 10.15 mg/mL in 1:1 EtOH/diH_2_O (1,2-dioleoyl-sn-glycero-3-phospho-L-serine or DOPS, Avanti, 840035) and added at a final concentration of 10 μM. The inhibitor was circulated in the bath solution after the equilibration period and prior to PE preconstriction.

### Pressure myography in lipid experiments.

For evaluation of PIP_2_ dilation, third-order mesenteric arteries were cannulated, pressurized to 80 mmHg, slowly warmed to 37°C, and equilibrated to myogenic tone. After myogenic tone plateaued and stabilized for a period of 10 minutes (D_EQ_), 10 μM diC8PIP_2_ (10 mM stock in Krebs-HEPES, Cayman Chemical, 64910) was added to the bath and circulated for 15 minutes in a total system volume of 10 mL. The maximum change in diameter was recorded within the respective incubation period (D_PIP2_). For experiments with Kir2.1 inhibitors, drug or lipid was added after myogenic tone developed and the diameter plateaued for 10 minutes. ML-133 and PS were circulated for 30 minutes, while Ba^2+^ was circulated for 15 minutes prior to adding exogenous diC8PIP_2_. Maximum change in diameter was recorded (D_PIP2_). Next, lipids and inhibitors were washed out of the system for 5 minutes. Arterial function was then evaluated by dilation to 1 μM NS309 and constriction to 30 mM KCl. As with other pressure myography experiments, Ca^2+^-free Krebs-HEPES was circulated through the system at the end of the experiment and diameter was recorded 10 minutes later (D_MAX_). Two zones were monitored for arterial dimeter, and the average of the 2 measurement are reported per artery. Zones were excluded if stable myogenic tone could not be achieved for the duration of the experiment. Lastly, if observable myogenic tone constriction occurred following a transient constriction or dilation to lipids, it was not recorded as the maximum change in diameter. For all experiments, time to maximum dilation is reported as the nearest half-minute. The dilation to diC8PIP_2_ was measured by the percent change before application of the lipid (Equation 4).

(Equation 4)
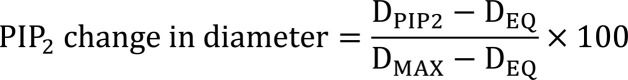


The data for maximum change in diameter for PS was taken from experiments where PS was circulating on the artery for 30 minutes prior to diC8PIP_2_ application (Equation 5).

(Equation 5)
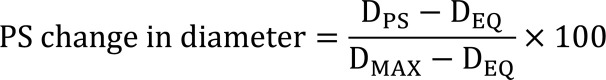


### Genomic DNA gel.

Genomic DNA was isolated from lung tissue from *Eln*^fl/fl^/Cre^–^ and *Eln*^fl/fl^/Cre^+^ mice between 10 and 12 weeks old. Lung samples were incubated at 56°C overnight in lysis buffer (20 mM Tris Base; 150 mM NaCl; 1 mM EDTA; 1 mM EGTA; 20 mM NaF; 0.5% Triton x-100) supplemented with 100 μg/mL of Proteinase K. Isopropanol was used to precipitate DNA, and ethanol was used to wash DNA. The dried DNA pellet was resuspended in autoclaved water and incubated for 1 hour at 56°C to allow the DNA to go into solution. The concentration was measured via nanodrop; then, 15 ng/μL DNA stocks were stored at –20°C until PCR was performed. The following primers were used to differentiate between WT and KO mice: WT F: 5′-CCATGTGGGTGCTGTAAGCT-3′, Excision R: 5′-GTGTGTGTAGCTGAGGAATGGG-3′, and LoxP Site R: 5′-CCTACCTTTCTGGGGCCACT-3′ (ordered from Integrated DNA Technologies). Each PCR reaction contained 10 μL of MyTaqRed Mix (Bioline, C755G95), 350 nM of each primer, and 100 ng of gDNA. Total reaction volume was 20 μL. Initial denaturation temperature was 95°C for 5 minutes, followed by 28 cycles of with 30 seconds denaturation, annealing, and elongation steps at 95°C, 61°C, and 72°C, respectively. The final annealing step was 72°C for 5 minutes. PCR products were run on a 1% agarose gel containing 0.01% ethidium bromide for 50 minutes at 120 V. Gels were imaged using a UV light source. The expected bands are 243 bp (WT), 283 bp (KO LoxP site), and 410 bp (excision product) (54).

### qPCR.

Mesenteric vasculature was trimmed of connective tissue, snap frozen in liquid nitrogen, and stored in –80°C. RNA isolation from tissue was achieved using an Aurum Total RNA Fatty and Fibrous Tissue Kit (Bio-Rad, 7326870). RNA yields were between 40 and 200 ng/μL. cDNA synthesis was next performed using SuperScript IV Reverse Transcriptase (Thermo Fisher Scientific, 18090050). Total well volume was 20 μL with equal amounts of cDNA for each sample loaded in 8μL, and master mix was 12μL, containing: (a) Taqman gene expression master mix (Thermo Fisher Scientific, 4369016), (b) probe of interest (Taqman probe Mm00514670_m1 *Eln* FAM-MGB, Thermo Fisher Scientific, 4453320), and an (c) in-well control (Taqman probe Mm00437762_m1 *B2M* VIC PL, Thermo Fisher Scientific, 4448485). Each sample was loaded in triplicate. Protocol was run using a Bio-Rad Thermal Cycler using a standard protocol for 40 cycles. Quantification was done using the ΔΔCt method, and Student’s *t* test was used for statistical analysis.

### En face quantification for hydrazide Eln^fl/fl^/Cre^–^ and Eln^fl/fl^/Cre^+^ mice.

ImageJ (NIH) was used for image analysis. The hydrazide channel was projected as a *Z* stack image, and brightness and contrast were adjusted for visualization of HIEL. Next, the Histogram Analysis method was used to quantify distribution of signal intensity. The cutoff for black pixels was determined by looking at the color-coded legend for the histogram. The number of black pixels per image was then calculated as the percentage of total pixels. Subtracting this number from 100 gives the percentage of pixels containing hydrazide signal. These numbers were plotted to determine hydrazide coverage in each image. Student’s *t* test was used for statistical analysis.

### En face quantification for PS distribution in en face view.

ImageJ was used for image analysis. The PS channel was thresholded, and “Analyze Particles” function was used to determine the area containing signaling and the number of puncta in the image, where particle size identification was set to be 0-Infinity. The results table in ImageJ contained the number of puncta and the coverage area of puncta. These values were normalized using the average value of control samples.

For heatmap analysis, the Royal Lookup Table in ImageJ was used to differentiate between low and high intensity signal. The index separating low- and high-intensity pixels was determined by looking at the color-coded legend for the histogram for the last blue bin. Empty pixels were defined as index 0 and were not included in the low-intensity category. The numbers of low- and high-intensity pixels were then normalized to the average value of control images.

### Statistics.

Data are presented as mean ± SEM unless otherwise noted. Several statistical tests are used throughout the manuscript and are specified for each experiment in figure legends and respective method sections. These tests include 2-tailed Student’s *t* test, Brown-Forsythe and Welch ANOVA, ordinary 1-way ANOVA, and repeated measures 2-way ANOVA. have been used for appropriate experiments. Post hoc analysis has been done where appropriate with Sidak’s multiple comparisons test, Holm-Sidak multiple comparisons, or Tukey’s multiple comparisons. *P* < 0.05 was considered to be statistically significant.

### Study approval.

The experiments within this manuscript were approved by the University of Virginia Animal Care and Use Committee.

### Data and materials availability.

All data needed to evaluate the conclusions in the paper are present in the paper and/or the supplementary materials.

## Author contributions

Conceptualization was contributed by CAR and BEI. Methodology was contributed by CAR, RP, MAL, YLC, MK, BAC, PJH, SMP, CJL, RPM, JEW, and SKS. Investigation was contributed by CAR, RP, and BEI. Visualization was contributed by CAR an BEI. Supervision was contributed by BEI. Writing of the original draft was contributed by CAR and BEI. Review and editing of the manuscript was contributed by All authors.

## Supplementary Material

Supplemental data

## Version 1 04/04/2023

In-Press Preview

## Version 2 05/08/2023

Electronic publication
