# Supplementary material for: Polarized localization of phosphatidylserine in the endothelium regulates Kir2.1
Source: JCI Insight. 2023 May 8;8(9):e165715. doi: 10.1172/jci.insight.165715 (PMC10243826; doi:10.1172/jci.insight.165715)
Supplement: Supplemental data [file jciinsight-8-165715-s203.pdf]

Supplemental Materials for

Polarized localization of phosphatidylserine in endothelium regulates Kir2.1

Claire A. Ruddiman, Richard Peckham, Melissa A. Luse, Yen-Lin Chen, Maniselvan Kuppusamy, Bruce Corliss, P. Jordan Hall, Chien-Jung Lin, Shayn M Peirce, Swapnil K. Sonkusare, Robert P. Mecham, Jessica E. Wagenseil, Brant E. Isakson*

*Corresponding author. Email: brant@virginia.edu

This PDF file includes:

Figs. S1 to S9

Tables S1 to S5

Supplemental Methods

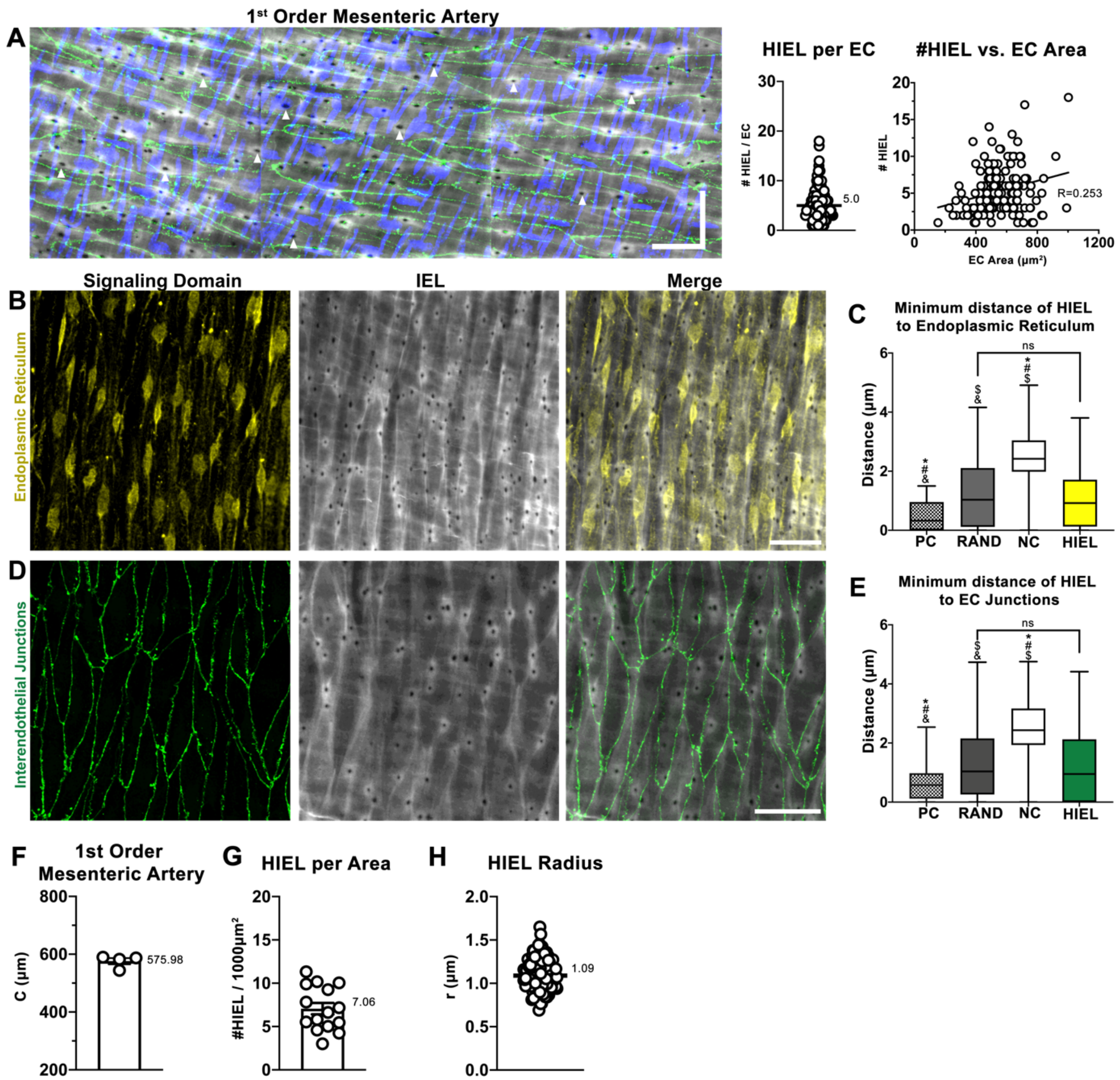

Supplemental Figure 1: HIEL are randomly distributed with respect to endothelial signaling hubs in first order mesenteric arteries. **(A)** Representative stitched confocal image of a first order mesenteric artery prepared en face and stained for nuclei (blue) via DAPI, the IEL (grey) via Alexa-Fluor 488-linked hydrazide, and interendothelial junctions (green) via claudin-5. Scale bar is 30µm in both directions. Quantification of HIEL per EC and correlation of HIEL per EC versus EC area. N=4 mice, n=4 arteries, n=12 ROIs, and n=155 ECs. (**B**) Representative en face confocal

image of endoplasmic reticulum (ER, yellow) detected via calnexin and IEL (grey), (**C**) box and whiskers plot of minimum distance of real-world HIEL centers to ER compared to Matlab-simulated HIEL centers. N=1 mouse, n=1 artery, n=2 ROIs, Area= 6.96×10^4 μm^2, and n=315 HIEL. (**D**) Representative en face confocal image of interendothelial junctions (green) detected via claudin-5 and IEL (grey), (**E**) box and whiskers plot of minimum distance of real-world HIEL centers to interendothelial junctions compared to Matlab-simulated HIEL centers. N=6 mice, and n=10 arteries, n=15 ROIs, Area=1.66×10^5 μm^2, and n=1200 HIEL. Statistical test: Brown-Forsythe and Welch ANOVA. # indicates a p <0.0001 significant difference to real-world HIEL distribution, * indicates a p <0.0001 significant difference to RAND, $ indicates a p <0.0001 significant difference to NC distribution, and & indicates a p <0.0001 significant difference to PC. (**F**) Circumference (C) of first order mesenteric arteries measured as the width of the artery in an en face preparation. N=4 mice, n=4 arteries. (**G**) HIEL per area taken from en face images that were used for HIEL spatial pattern analysis with respect to claudin-5. Number of HIEL was determined via the in-house Matlab program. (**H**) HIEL radius measurements obtained from in-house Matlab program.

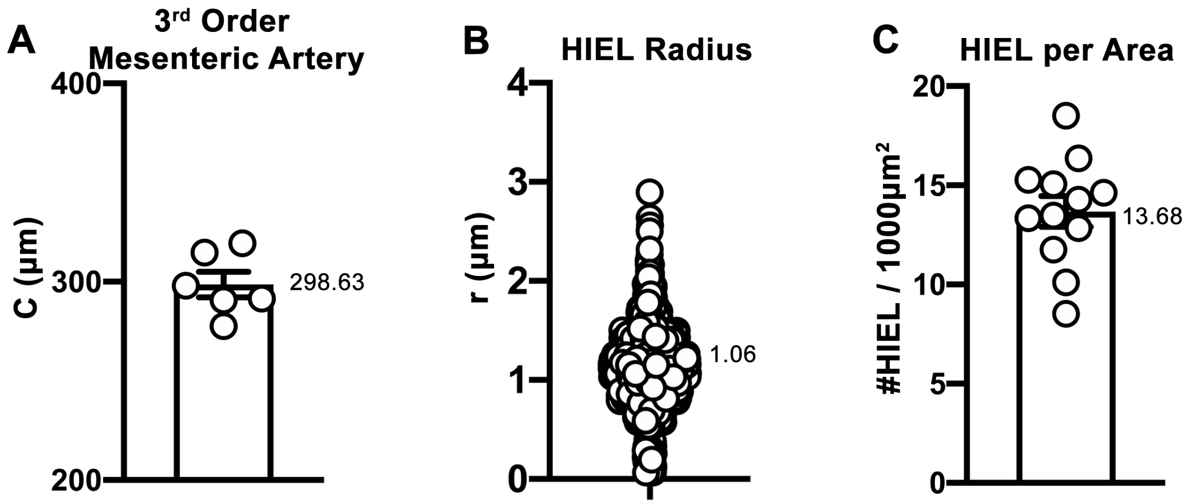

Supplemental Figure 2: Quantitative data obtained from third order mesenteric artery en face preparations. (**A**) Circumference of third order mesenteric arteries measured as the width of the artery in an en face preparation. N=5 mouse, n=6 arteries. (**B**) HIEL radius measurements obtained from in-house Matlab program. N=6 mice, and n=10 arteries, n=22 ROIs, Area=1.48x10^5µm^2, and n=2166 HIEL. (**C**) HIEL per area taken from en face images used in Matlab analysis. Number of HIEL was determined via the in-house Matlab program. N=4 mice, n=4 arteries, n=12 ROIs, and Area=1.67x10^5 µm^2.

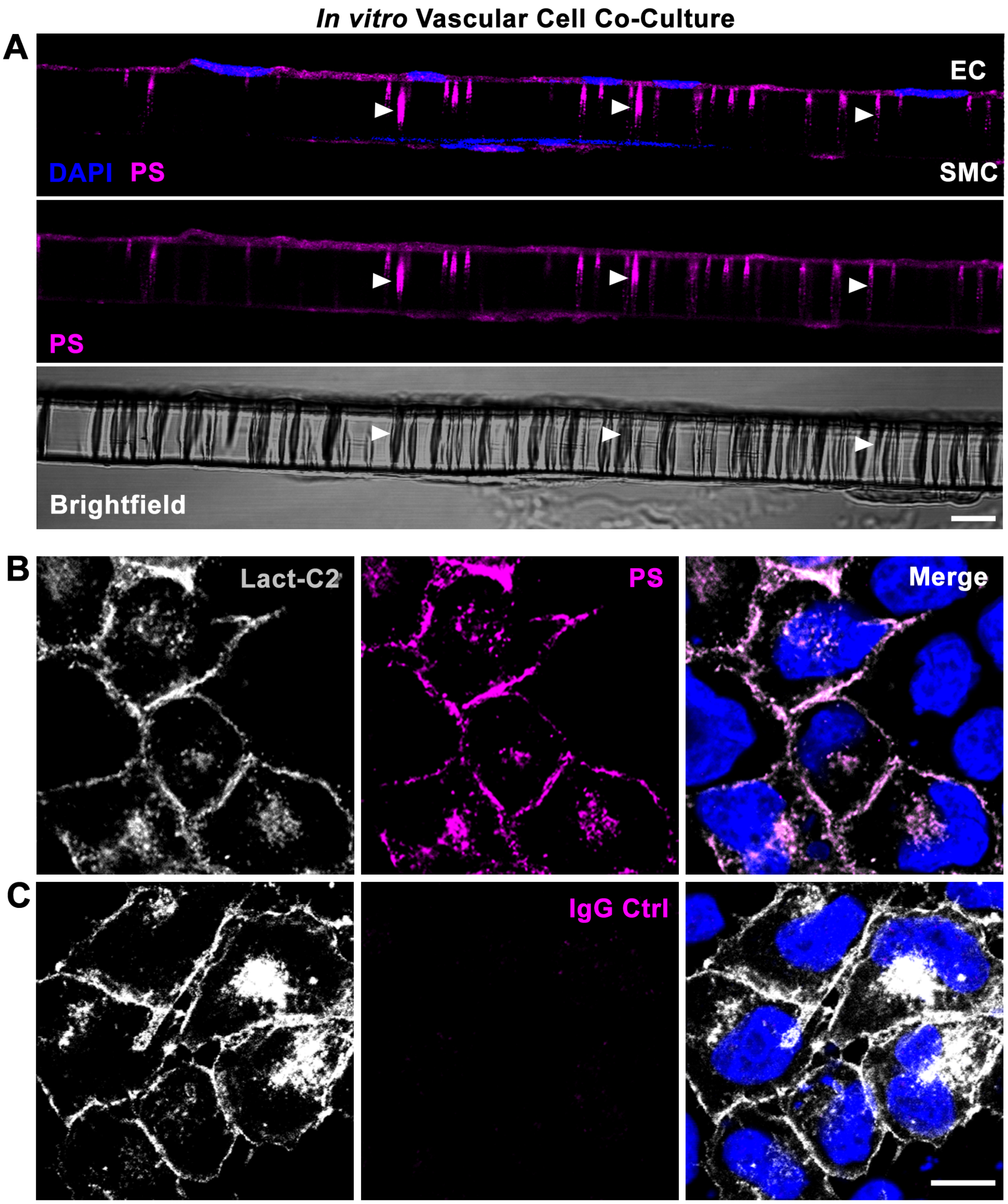

Supplemental Figure 3: Validation of PS antibody. **(A)** Transverse cross-sections of an in vitro vascular cell co-culture model where EC and SMC are plated on either side of a Transwell. Nuclei (blue) are detected via DAPI and PS (magenta). Brightfield image of Transwell is shown in the bottom panel. Arrowheads indicate PS localization to in vitro MEJs. (**B**) HeLa cells transfected

with Lact-C2-GFP plasmid (white) and co-stained with PS antibody or (**C**) IgG control. Nuclei (blue) are detected via DAPI. Scale bars are 10μm.

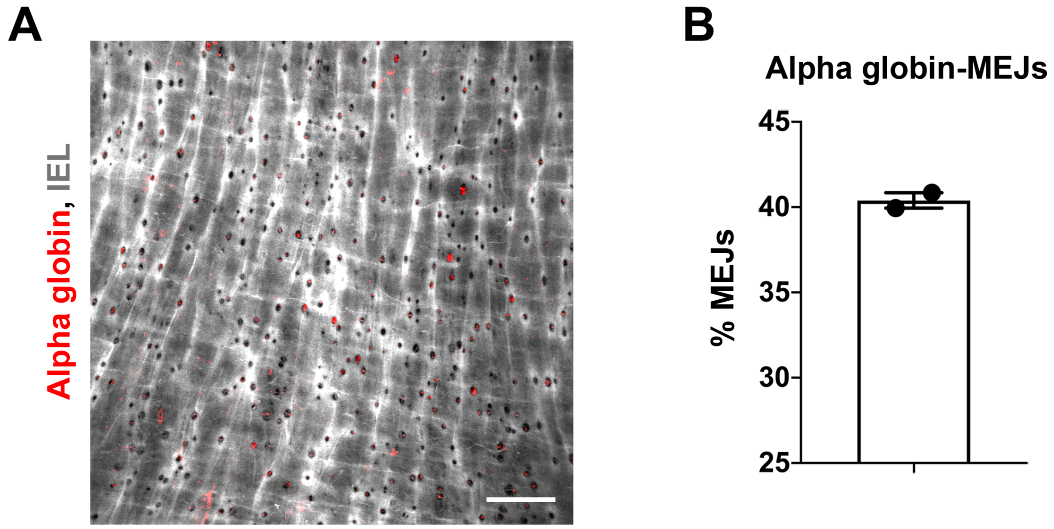

Supplemental Figure 4: Quantification of hemoglobin alpha at the MEJ. (A) Representative en face image of a third order mesenteric artery to detect the IEL (grey) and alpha globin (red). (**B**) Quantification of alpha globin in MEJs (n=2 mice, 2 arteries). Scale bar is 30µm.

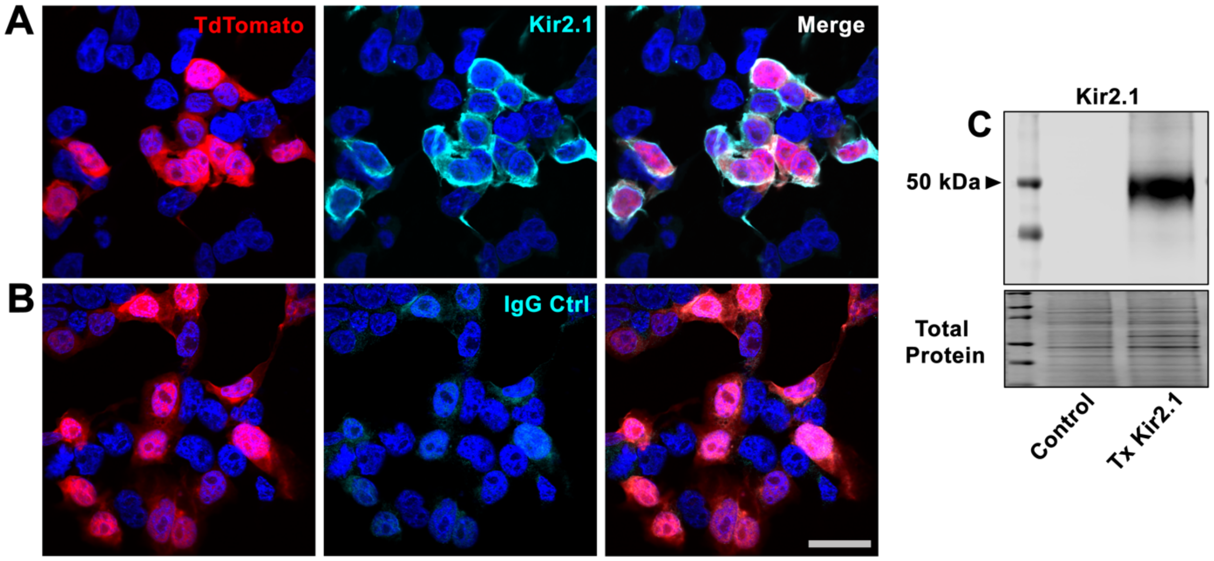

Supplemental Figure 5: Validation of Kir2.1 antibody. (**A**) HEK293T cells transfected with Kir2.1-T2A-tdTomato plasmid (red) and co-stained with Kir2.1 antibody or (**B**) IgG control. Nuclei (blue) are detected via DAPI. Scale bar is 30µm. (**C**) Western blot detection of Kir2.1 protein in HEK293T cells that were untreated or transfected with Kir2.1-T2A-tdTomato plasmid. Total protein was used as a loading control.

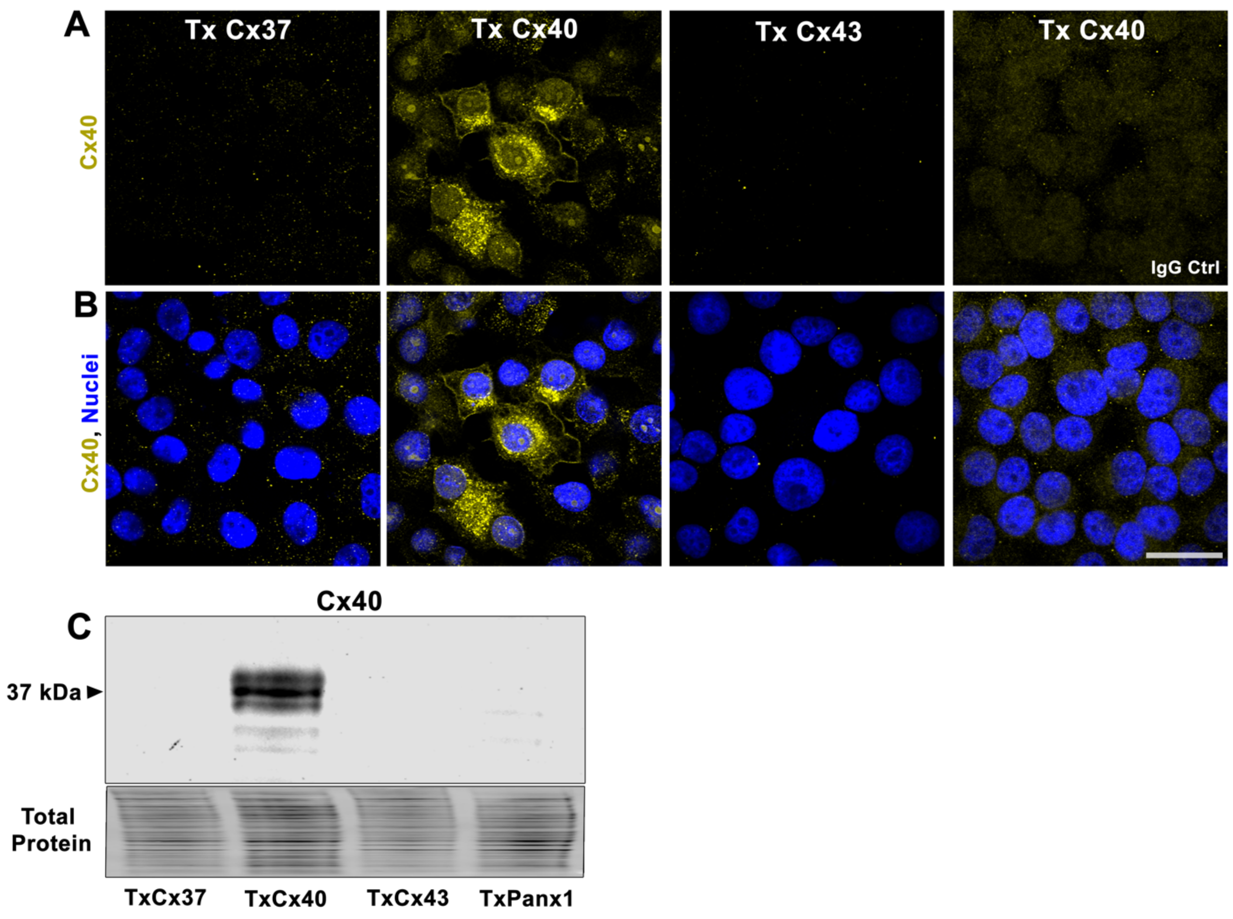

Supplemental Figure 6: Validation of Cx40 antibody. (**A**) HeLa cells transfected with a connexin plasmid, either connexin 37 (Tx Cx37), connexin 40 (Tx Cx40), or connexin 43 (Tx Cx43), then stained using a Cx40 antibody or IgG control to evaluate Cx40 antibody specificity. Nuclei (blue) are detected via DAPI. Scale bar is 30µm. (**C**) Western blot detection of Cx40 protein in HeLa cells that were transfected with plasmids for Cx37, Cx40, Cx43, and Panx1. Total protein was used as a loading control.

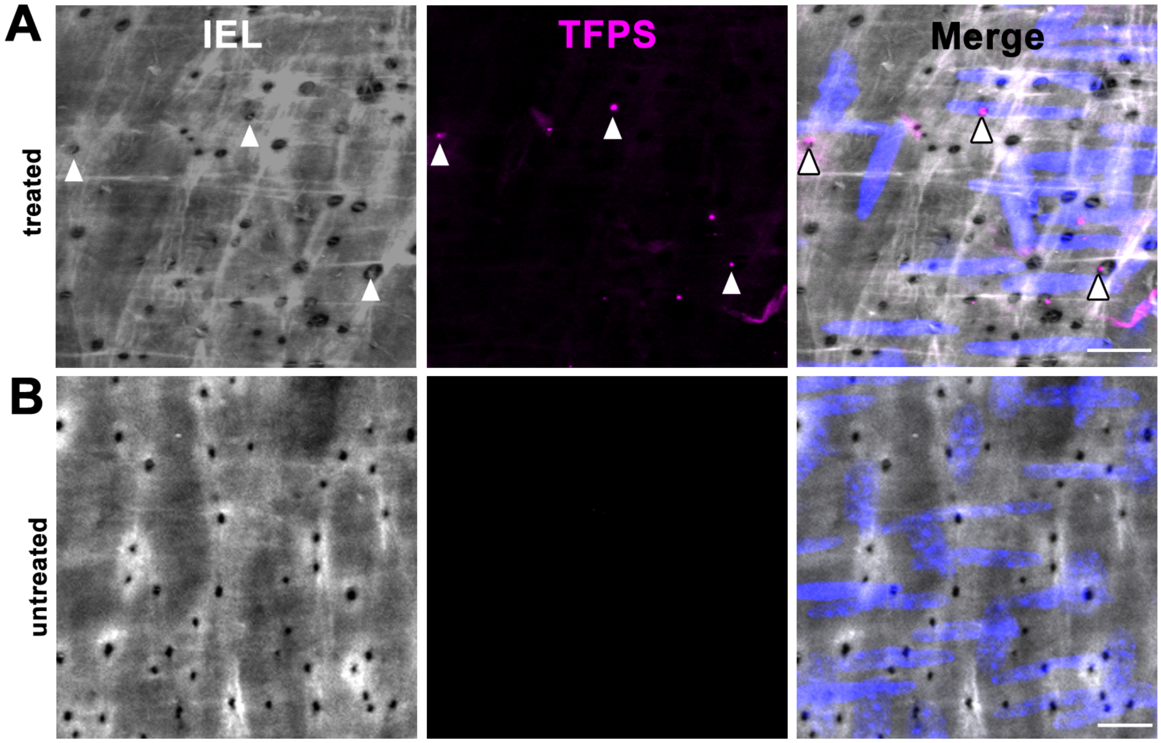

Supplemental Figure 7: Exogenous application of TopFluor-PS localizes to the MEJ in intact third order mesenteric arteries. Live third order mesenteric arteries prepared en face after treatment with (**A**) 10µM TopFluor-PS in the bath solution of a pressure myography setup or (**B**) without treatment. Nuclei (blue) are detected via DAPI and IEL (grey) is detected via Alexa Fluor linked hydrazide. Arrowheads indicate TopFluor-PS localization to MEJ. Scale bars are 10µm.

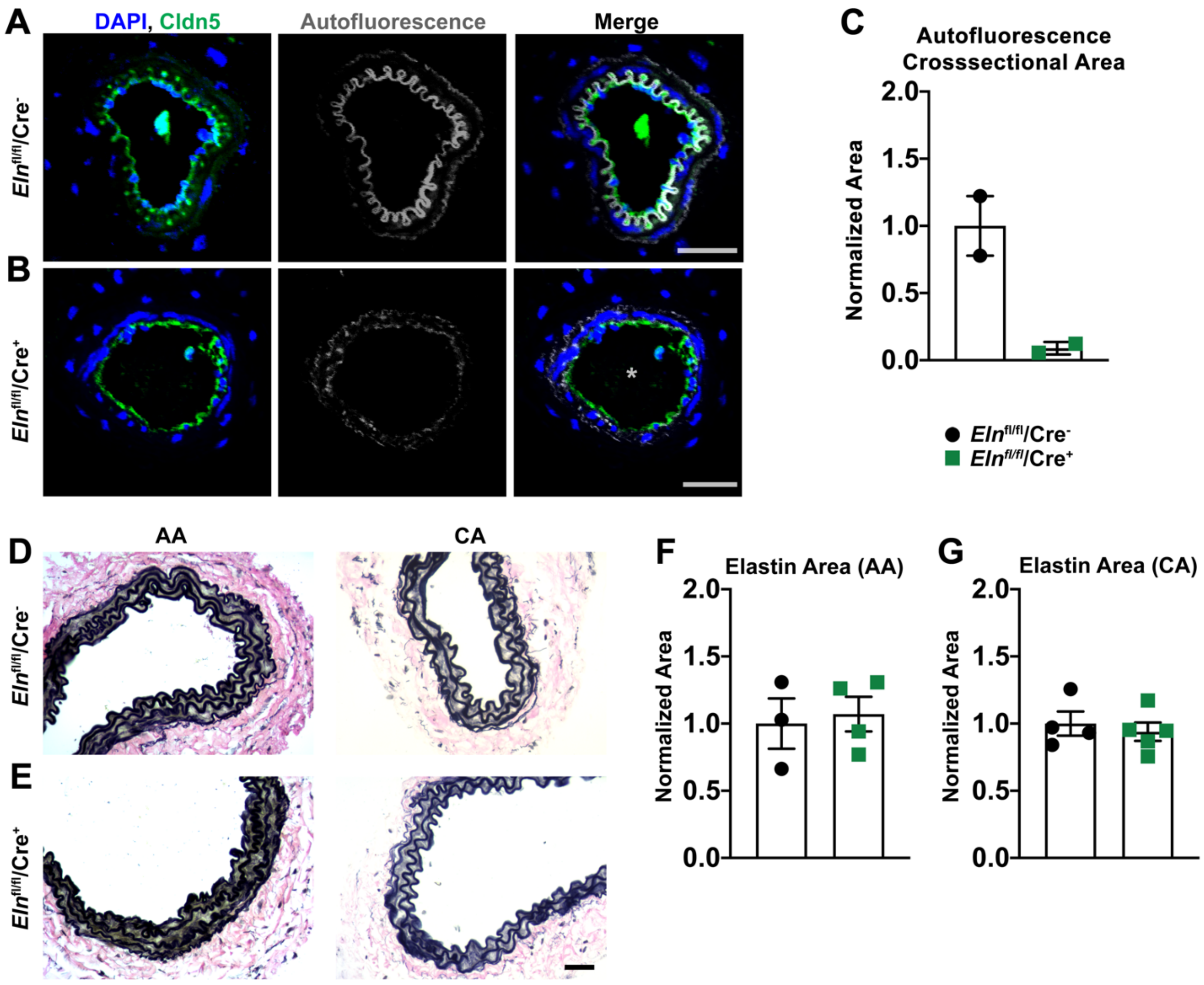

Supplemental Figure 8: EC-specific knockout of elastin disrupts IEL in resistance arteries and not large conduit arteries. Representative images of cross-sections from third order mesenteric arteries taken from (**A**) $Eln^{fl/fl}$/Cre^- and (**B**) $Eln^{fl/fl}$/Cre^+ mice where nuclei (blue) are detected via DAPI, IEL (grey) is detected via autofluorescence, and interendothelial junctions (green) are detected via claudin-5. Scale bar is 30μm. N=2 mice per group. (**C**) Quantification of cross-sectional IEL area detected via autofluorescence. Representative images of cross-sections from the abdominal aorta (AA) and carotid artery (CA) of (**D**) $Eln^{fl/fl}$/Cre^- and (**E**) $Eln^{fl/fl}$/Cre^+

mice. Scale bar is 30µm. Quantification of Verhoeff stain in (**F**) AA and (**G**) CA. N= 3-5 mice per group. Student's t-test.

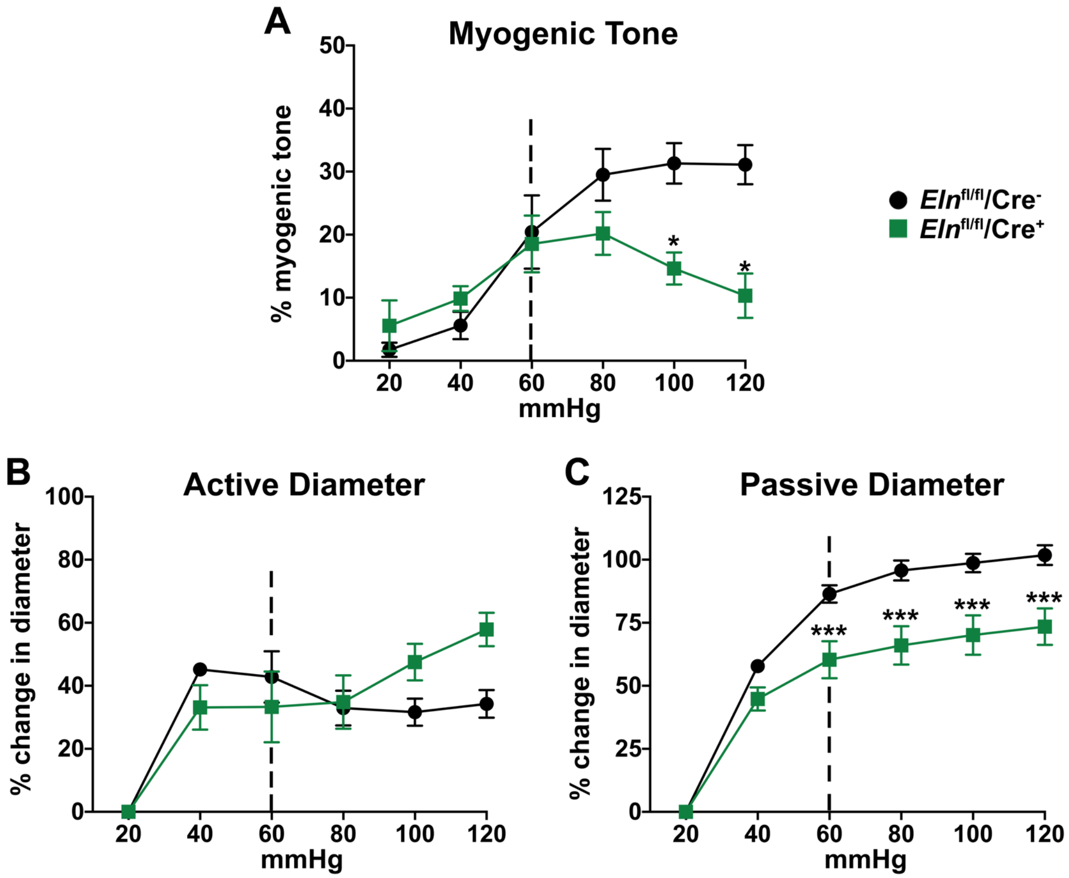

Supplemental Figure 9: Determination of optimal pressure for myography experiments on *Eln*$^{fl/fl}$/Cre^{+} mice. Pressure myography experiments on third order mesenteric arteries from *Eln*$^{fl/fl}$/Cre^{-} and *Eln*$^{fl/fl}$/Cre^{+} mice, where (**A**) is myogenic tone, (**B**) is the active diameter, and (**C**) is the passive diameter. Dotted line indicates the optimal pressure for *Eln*$^{fl/fl}$/Cre^{+} arteries. N=3 mice per group, n=4-6 arteries per group. A repeated measures two-way ANOVA was performed, with $p<0.0001$ significant difference between genotypes for Myogenic Tone and Passive Diameter analyses. A Sidak's multiple comparison post hoc analysis was performed at each pressure where * $p<0.050$ and *** $p<0.001$. No significant difference was detected for Active Diameter analysis.

		Figure 1D	Figure 1F	Figure 1H	Figure 3F
**PC**	Minimum	0	0	0	0
	1st quartile	0	0	0	0
	Median	0.12	0.33	0.46	0.12
	3rd quartile	0.77	0.98	0.93	1.14
	Maximum	4.32	4.705	3.82	1.50
**RAND**	Minimum	0	0	0	0
	1st quartile	2.12	0.12	0.23	0.23
	Median	5.24	1.09	1.04	1.16
	3rd quartile	8.90	2.19	2.08	1.98
	Maximum	30.96	6.00	6.38	3.85
**NC**	Minimum	0	0	0	1.52
	1st quartile	2.32	1.98	1.86	1.82
	Median	3.12	2.42	2.32	2.19
	3rd quartile	3.84	3.04	3.00	2.76
	Maximum	14.45	4.91	4.50	4.17
**HIEL**	Minimum	0	0	0	0
	1st quartile	2.14	0.23	0.15	0
	Median	5.40	0.92	0.99	0.61
	3rd quartile	9.08	1.85	2.08	1.97
	Maximum	31.78	6.11	5.47	3.34

Supplemental Table 1: Reported values for box and whiskers plots for Matlab analysis of en face images.

Variable	Value	Unit	Source	Description
**C**	298.6	µm	N=4, n=6 arteries **Fig. S2A**	Circumference of 3rd order mesenteric arteries
**C_{IEL}**	298.6	µm	Assume that it is equal to C	Circumference of IEL
**d**	2.1	µm	N=5 mice, n=2166 HIEL **Fig. S2B**	Average diameter of HIEL
**$\bar{A}_{xy}$**	13,942.9	μm^2	Experimental parameter	Area of en face images
**$\tilde{N}_{HIEL}$**	190.7	HIEL / image	N=4, n=4 arteries, n=12 images **Fig. S2C**	Average number of HIEL
**ρ_{HIEL}**	13.6	HIEL per $1000\mu m^2$	**Eq. 1**	Density of HIEL per $1000\mu m^2$

Supplemental Table 2: Summary of quantitative data obtained from en face images. Measurements taken from third order mesenteric arteries, either manually, automatically via in-house Matlab program, or calculated from direct measurements.

Variable	Value	Units	Source	Description
**Y_{TEM}**	0.070	µm	Experimental parameter	Thickness of transverse TEM sections
**$A_{TEM,1}$**	20.902	μm^2	**Eq. 2**	Artery area spanning **Y_{TEM}**
**$A_{TEM, d}$**	627.06	μm^2	**Eq. 4**	Artery area spanning the length **d**
**L_{IEL}**	1791.6	µm	**Eq. 5**	Length of IEL across 6 TEM sections
**$HIEL_{TEM}$**	9	HIEL per A_{TEM}	**Eq. 6**	Number of HIEL expected across $A_{TEM,d}$

Supplemental Table 3: Calculated parameters of arterial cross-sections imaged via TEM. Experimental and calculated parameters for converting en face quantitative data to be interpretable in a TEM geometry.

Case Definition	Variable	Value	Units	Equation	Description
**Case1: <u>Maximum case</u>, assumes HIEL are evenly distributed**	$D_{HIEL,\ E}$	54	TEM detections	**Eq. 7**	HIEL detections over 6 sections for Case 1
**Case 2: Assumes HIEL are <u>randomly</u> distributed**	$D_{HIEL,\ R}$	32		**Adjusted Eq. 7 (see text)**	HIEL detections over 6 sections for Case 2
**Case 3: <u>Minimum case</u>, represents the <u>rare</u> case where each HIEL only appear on 1/6 of sections**	$D_{HIEL,\ S}$	9		**Adjusted Eq. 7 (see text)**	HIEL detections over 6 sections for Case 3
**Normalized Case 1**	$D_{HIEL,\ EN}$	30	TEM detections <u>per 1000µm IEL</u>	**Eq. 8**	HIEL detections over 6 sections for Case 1 normalized to IEL length
**Normalized Case 2**	$D_{HIEL,\ RN}$	17.8		**Eq. 9**	HIEL detections over 6 sections for Case 2 normalized to IEL length
**Normalized Case 3**	$D_{HIEL,\ SN}$	5		**Eq. 10**	HIEL detections over 6 sections for Case 3 normalized to IEL length

Supplemental Table 4: Case scenarios that consider the potential spatial distributions of HIEL. Prediction of HIEL incidence in TEM sections with consideration of spatial distributions of evenly distributed (**Case 1**), randomly distributed (**Case 2**), or sparsely distributed (**Case 3**). Predictions are then normalized to obtain ratios with respect to IEL length, a metric that can be reproducibly measured in TEM images (**Normalized Cases 1-3**).

	Experimental Membrane Voltages		
**Pipette Solution**	**-140mV**	**-30mV**	**-20mV**
No Treatment	-27.55 ± 3.70	2.92 ± 0.74	3.78 ± 0.80
10µM PIP_2	-42.51 ± 5.77	5.59 ± 0.36	7.00 ± 0.31
10µM PS only	-43.95 ± 5.18	10.77 ± 0.54	11.95 ± 0.54
10µM PIP_2 + 10µM PS	-38.207 ± 10.79	2.08 ± 0.86	3.66 ± 1.18

Supplemental Table 5: Comparison of average, basal whole-cell Kir2.1 current in HEK293T cells across different membrane voltages. N=5-7 cells per group.

Supplemental Methods

Predicting the incidence of myoendothelial junctions in transmission electron microscopy cross-sections

First, we considered basic descriptive data obtained from en face images (Supplemental Table 2) including the circumference of the artery (**C**, Supplemental Figure 2A) the average diameter of HIEL (**d**, Supplemental Figure 2B), and spatial density of HIEL ($\boldsymbol{\rho_{HIEL}}$, Supplemental Figure 2C), which was calculated using Equation 1.

$$\rho_{HIEL} = \frac{\tilde{\mathrm{N}}_{\mathrm{HIEL}}}{\bar{\mathrm{A}}_{\mathrm{xy}}} \qquad (1)$$

$\boldsymbol{\tilde{N}_{HIEL}}$ and **d** were quantified via the in-house Matlab program, and **C** was measured as the width of the artery when prepared en face. For this calculation, we assume the measured circumference of the artery is equivalent to the circumference of the IEL ($\mathbf{C_{IEL}}$).

Next, we considered the transverse view of the artery when prepared for TEM imaging (Supplemental Table 3). The TEM section is a thin cross section sliced from a third order mesenteric artery where the thickness of an individual section is $\mathbf{Y_{TEM}}$. Since our quantification of HIEL thus far is from the en face view, we wanted to quantify the IEL en face surface area in 1 TEM section in order to predict the density of HIEL in TEM sections. A single TEM cross-section corresponds to an en face surface area of 20.902μm^2, which was calculated using Equation 2.

$$A_{TEM,1} = Y_{TEM} \times C_{IEL} \quad (2)$$

An important note is **d** is much larger than $\mathbf{Y_{TEM}}$, such that the average HIEL will span 30 TEM sections Equation 3.

$$\text{TEM sections} = \frac{d}{Y_{TEM}} \quad (3)$$

For conceptual clarity, we will consider 6 TEM sections equally spaced apart along artery length **d**, which corresponds to an IEL surface area of 627.06μm^2 Equation 4 and an IEL length of 1791.6μm Equation 5.

$$A_{TEM,d} = C_{IEL} \times d \quad (4)$$

$$L_{IEL} = 6 \times C_{IEL} \quad (5)$$

Using $\boldsymbol{\rho_{HIEL}}$ and $\mathbf{A_{TEM,d}}$, we calculated we would detect a total of 9 unique HIEL across a sectioned artery length of **d** with Equation 6.

$$HIEL_{TEM} = \frac{\rho_{HIEL} \times A_{TEM,d}}{1000\mu m^2} \quad (6)$$

The final variable we considered in our prediction is the spatial distribution of holes throughout the IEL. For this, we considered three cases of distribution (Supplemental Table 4): (1) uniform distribution (maximum detection), (2) random distribution, and (3) sparse distribution (minimum detection).

For Case 1, we considered 6 TEM sections over artery length **d** and assumed each of the 9 HIEL were exactly aligned with the start and end of the sectioned area ($\mathbf{HIEL_{TEM,6E}}$) such that the HIEL distribution is uniform, and the number of HIEL detections by the microscope user is 54 (Equation 7).

$$D_{\mathrm{HIEL,E}} = 6 \times \mathrm{HIEL_{TEM,6E}} \tag{7}$$

However, due to the nature of TEM, the entire cross-sectional area cannot be imaged due to visibility blockages by grid lines, indicating all 54 HIEL cannot be imaged by the user, even if they were present. In order to account for this technical challenge, the predicted number of HIEL detections can be normalized to the length of IEL in the TEM image. This normalization process results in a numerical value with the units of HIEL per length of IEL. **Thus, if HIEL are uniformly distributed, then we expect 30 HIEL per 1000μm IEL length** (Equation 8).

$$\mathrm{D_{HIEL,EN}} = \frac{\mathrm{D_{HIEL,E}}}{\mathrm{L_{IEL}}} \times 1000\mu\mathrm{m} \tag{8}$$

However, based on the Matlab simulations (Figure 1), we have shown HIEL are randomly distributed. We therefore incorporated a random distribution of HIEL into our prediction. Thus,

instead of assuming each HIEL would have 6 detections across each of the 6 sections, we assumed each HIEL had a different number of detections due to a random alignment with the beginning and end of the length sectioned area. The random distribution of detections was considered as follows**:** 2 HIEL with 6 detections each (12 detections), 1 HIEL with 5 detections each (5 detections), 2 HIEL with 4 detections each (8 detections), 1 HIEL with 3 detections each (3 detections), 1 HIEL with 2 detections each (2 detections), and 2 HIEL with 1 detection each (2 detections), bringing the total to 32 detections ($D_{HIEL,R}$, adjusted **Equation 7** random distribution). Using the same normalization process as described above, we determined **if HIEL are randomly distributed, we expect 17.8 HIEL per 1000µm length of IEL** (Equation 9).

$$D_{HIEL,RN} = \frac{D_{HIEL,R}}{L_{IEL}} \times 1000\mu m \tag{9}$$

Due to the random distribution of HIEL, there are some areas of IEL with relatively few HIEL. Thus, a third case is considered to reflect a sparse distribution scenario. In this minimum case scenario, each of the 9 HIEL are only aligned with 1 TEM section and thus is only detected once during imaging. For 9 HIEL considered across 6 sections, with each HIEL appearing in only 1 section, there are 9 total detections ($D_{HIEL,S}$, adjusted **Equation 7** random distribution). Normalizing these to the IEL within TEM images as for the previous two scenarios, **5 HIEL are expected per 1000µm length of IEL** (Equation 10).

$$D_{HIEL,SN} = \frac{D_{HIEL,S}}{L_{IEL}} \times 1000\mu m \tag{10}$$

Since we have demonstrated the HIEL are randomly distributed, and there are areas sparse in HIEL, **the predicted range of HIEL density in the TEM images is 5-17.5 HIEL per 1000µm IEL analyzed**.

Vascular cell co-culture

Vascular cell co-culture (VCCC) was created with human coronary artery smooth muscle cells (HCoASMCs) and human umbilical vein endothelial cells (HUVECs), paraffin embedded, and stained as originally described by us. (7, 69) Cell lines were purchased from ATCC and use before 10 passages.

Transfections for immunohistochemistry

HeLa or HEK293T were plated in 6-well dishes and grown until 70–80% confluent. HeLa cells were obtained from ATCC. HEK293T cells were a kind gift from Dr. Douglas Bayliss (University of Virginia, Department of Pharmacology, Charlottesville, VA). Each well contained a 10mm circular glass coverslip at the bottom. Transfections were performed either with Lipofectamine 3000 or with the Lonza Nucleofector kit for HeLa cells. For validation of PS antibody, HeLa cells were plated in a 12-well dish and transfected for 17 hours with 1.8µg plasmid, 3.65µl P3000, and 2.7µl Lipofectamine 3000 per well. For validation of Kir2.1 antibody, HEK293T cells were plated in a 6-well dish and transfected for 24 hours with 2.25µg plasmid, 4.48µl P3000, and 3.47µl Lipofectamine 3000 per well. For validation of Cx40 antibody, HeLa cells were transfected using nucleofection (Lonza, VCA-1001) where $1x10^6$ cells were transfected with 0.50µg plasmid and split across two wells of a 12-well dish.

For all validation experiments, cells were fixed with 4% PFA for 10 minutes at 4°C, washed with PBS 3x 5 minutes, then blocked for 1 hour with 1% fish skin gelatin, 0.20% Triton-X 100, 0.50% BSA, and 5% animal serum in PBS. Primary antibody was diluted in blocking solution at a 1:100 concentration overnight at 4°C. The next day, following 3x 5-minute PBS washes, secondary antibody incubations were performed at a concentration of 1:500 for 1 hour at RT with gentle shaking. After secondary incubations, samples were washed 1x with PBS for 10 minutes, then incubated with DAPI at a concentration of 1:5000 for 10 minutes in PBS, and washed 1x for 10 minutes with DAPI prior to mounting the coverslips onto microscope slides. Images were obtained using an Olympus FV1000 confocal microscope.

TopFluor PS experiments

Third order mesenteric arteries were dissected from male C57Bl6/J mice and secured to Sylgard squares using tungsten wire. Arteries were place in a 1.5ml Eppendorf tube containing Krebs-HEPES supplemented with 2mM Ca^{2+}. Next, arteries were incubated with DAPI at 16.67 µg/ml and Alexa Fluor 647-linked hydrazide at 3.3µM in Krebs-HEPES supplemented with 2mM Ca^{2+} for 30 minutes in a 37°C water bath. The artery was then cannulated on glass canula on a pressure myograph setup. After equilibration to 80mmHg, 10µM TopFluor-PS (Avanti, 810283P) was added to the bath solution and circulated for 30 minutes. The artery was then immediately prepared en face and secured to a microscope slide with a droplet of Prolong Gold with DAPI and a glass coverslip (no fixation step). Arteries were imaged on an Olympus Fluoview 1000 microscope.

VVG stain and quantification

Arteries were isolated from mice and fixed with 4% PFA at 4°C overnight, embedded in 3% agarose/PBS, then sent to the UVA Research Histology Core to be embedded in paraffin wax cut into 5μm cross-sections. The cross-sections were deparaffinized by heating to 60°C for 1 hour in a glass slide holder, followed by dehydration with Histoclear (National Diagnostics, HS-200) and a decreasing ethanol gradient. The Elastic Stain Kit (Verhoeff Van Gieson / EVG Stain) kit was purchased from Abcam (ab150667). Slides were placed into elastic stain solution for 15 minutes and rinsed with cool, running diH_2O to remove excess stain. Sections were differentiated by dipping the slides in 2% ferric chloride solution 15 times. The differentiation reaction was stopped by rinsing slides in cool, running diH_2O. Excess stain was further removed by dipping the slides in the sodium thiosulfate solution for 1 minute, followed by rinsing in cool, running diH_2O. The slides were then placed in Van Gieson solution for 2 minutes for a counterstain. Excess stain was removed by rinsing in two changes of 95% EtOH, followed by a 2-minute incubation in 100% EtOH. The slides were then quickly mounted using VectaMount media and a coverslip. Images were taken using a traditional light microscope and a 20x or 40x objective.

ImageJ was used for image analysis. The stained arterial cross-section image was opened in ImageJ. The colors of the original image were then separated by "H PAS stain" color deconvolution (Image → Color → Color Deconvolution → H PAS). The indigo image was analyzed because it specifically contained the Verhoeff elastin stain. The indigo imaged was converted to a black and white mask, and the threshold of indigo was automatically determined by the ImageJ software (Process → Binary → Convert to Mask). Student's t test.
